# Safety of [^177^Lu]Lu-NeoB treatment: a preclinical study characterizing absorbed dose and acute, early, and late organ toxicity

**DOI:** 10.1007/s00259-022-05926-2

**Published:** 2022-08-11

**Authors:** Eline A. M. Ruigrok, Marjolein Verhoeven, Mark W. Konijnenberg, Erik de Blois, Corrina M. A. de Ridder, Debra C. Stuurman, Luisa Bertarione, Katia Rolfo, Marion de Jong, Simone U. Dalm

**Affiliations:** 1 Dept. of Radiology and Nuclear Medicine, Erasmus MC, Erasmus University Medical Center, 3015 GD Rotterdam, The Netherlands; 2 Dept. of Experimental Urology, Erasmus MC, Erasmus University Medical Center, Rotterdam, The Netherlands; 3 Advanced Accelerator Applications, a Novartis Company, Colleretto Giacosa, Italy

**Keywords:** GRPR, [^177^Lu]Lu-NeoB, Radiotoxicity, Dosimetry, Radionuclide therapy

## Abstract

**Purpose:**

The radiolabeled gastrin-releasing peptide receptor (GRPR)-targeting antagonist NeoB is a promising radioligand for imaging and therapy of GRPR-expressing malignancies. In the current study, we aimed to discover the target organs of toxicity and the radiotoxic effects to these organs, when repeated dosages of [^177^Lu]Lu-NeoB are administered to healthy female and male mice.

**Methods:**

Animals received either 3 injections, with a 7-day interval, of vehicle (control group 1), 1200 pmol [^175^Lu]Lu-NeoB (control group 2) or 40 MBq/400 pmol, 80 MBq/800 pmol, and 120 MBq/1200 pmol [^177^Lu]Lu-NeoB (treatment groups 1, 2, and 3, respectively). At week 5, 19, and 43 after the first injection acute, early, and late organ toxicity, respectively, was determined. For this, histopathological and blood analyses were performed. To correlate the observed toxicity to absorbed dose, we also performed extensive biodistribution and dosimetry studies.

**Results:**

The biodistribution study showed the highest absorbed doses in GRPR-expressing pancreas, the liver, and the kidneys (the main organs of excretion). Both control groups and almost all animals of treatment group 1 did not show any treatment-related toxicological effects. Despite the high absorbed doses, no clear microscopic signs of toxicity were found in the pancreas and the liver. Histological analysis indicated kidney damage in the form of hydronephrosis and nephropathy in treatment groups 2 and 3 that were sacrificed at the early and late time point. In the same groups, increased blood urea nitrogen levels were found.

**Conclusion:**

In general, repeated administration of [^177^Lu]Lu-NeoB was tolerated. The most significant radiotoxic effects were found in the kidneys, similar to other clinically applied radioligands. The results of this study underline the potential of [^177^Lu]Lu-NeoB as a promising option for clinical therapy.

**Supplementary Information:**

The online version contains supplementary material available at 10.1007/s00259-022-05926-2.

## Introduction

Following the positive impact of radiolabeled somatostatin analogue peptides on neuroendocrine tumor patient care, there has been an increasing interest in the development of radioligands for diagnosis and therapy of other cancers [1]. High target expression is fundamental for successful visualization and effective cancer treatment. The gastrin-releasing peptide receptor (GRPR), also known as bombesin receptor subtype 2, is a G-protein coupled receptor that is overexpressed in several solid cancers, such as breast, lung, and prostate cancer [2–4]. These cancer types have high incidence and mortality rates indicating the need for novel treatment options [5].

Over the years, multiple GRPR-targeting ligands have been developed that were mostly based on the amphibian tetradecapeptide bombesin or the mammalian gastrin-releasing peptide [6]. The first generation of radiolabeled GRPR-targeting ligands were agonists, but a switch was made to antagonists when clinical studies reported gastrointestinal side effects as a result of GRPR activation [7]. Besides a better safety profile, radioantagonists often possess more favorable pharmacokinetics, i.e., faster clearance from background tissues and better tumor binding [8]. When a ligand is labeled with β^+^- and γ-emitters (e.g., Ga-68, In-111), it can be used for PET and SPECT imaging, respectively, and with β^−^- and α-emitters (e.g., Lu-177, Bi-213) for treatment. In this way, the same molecule can be used for both diagnostic imaging and for therapy, or so-called theranostics [9].

The potent GRPR-directed radioantagonist DOTA-p-aminomethylaniline-diglycolic acid-DPhe-Gln-Trp-Ala-Val-Gly-His-NH-CH[CH2-CH(CH3)2]2 (formerly known as NeoBOMB1, further referred to as NeoB) has been extensively evaluated in both preclinical and clinical studies [10–13]. Previous research has demonstrated a high GRPR binding affinity of NeoB, independent of the radiometal used, making it a promising candidate for theranostic applications [13]. Biodistribution studies with the Ga-68/Lu-177-labeled NeoB theranostic pair in mice bearing GRPR-expressing human prostate cancer PC-3 xenografts showed a favorable in vivo stability and high tumor uptake and retention [12]. Moreover, a preclinical in vivo efficacy study where PC-3 tumor-bearing mice were treated with 30 MBq/300 pmol, 40 MBq/400 pmol, or 60 MBq/600 pmol [^177^Lu]Lu-NeoB showed that all treatment groups had a significant delay in tumor growth compared to the control group [14]. In addition, a small first in human study in 4 prostate cancer patients with [^68^Ga]Ga-NeoB as a radioactive diagnostic agent for PET/CT was performed. High-contrast imaging allowed the detection of both primary and metastatic lesions [13]. On route to the implementation of NeoB theranostics into clinical practice, early phase clinical trials are currently ongoing [10, 15].

A potential concern for targeted radioligand therapy with β^−^- and α-emitters, at therapeutic dose levels, is the high accumulation of the radioligand in the kidneys as a result of renal clearance. Additionally, potential adverse effects may occur in organs with high physiological expression of the target, such as the GRPR-expressing pancreas in the case of [^177^Lu]Lu-NeoB, or in known radiosensitive tissues (e.g., the bone marrow and the reproductive tract) [12]. The dose limit for the pancreas has not been investigated in detail as it was not previously considered to be an organ at risk in the case of peptide receptor radionuclide therapy (PRRT) using the extensively studied radiolabeled octreotide derivatives.

The aim of this study was to investigate whether, and at what dose, (radio)toxicity occurs in healthy organs when a repeated dose treatment regime with [^177^Lu]Lu-NeoB is administered in an animal model. We have therefore administered three different radiation dosages (low, intermediate, high) of [^177^Lu]Lu-NeoB for which we characterized acute, early, and late organ toxicity. We also performed extensive biodistribution and dosimetry studies to correlate the absorbed organ doses with any observed toxicities. To our knowledge, this study is the first to report on the long-term toxicity of a repeated dose regimen of [^177^Lu]Lu-NeoB and is therefore indispensable for further clinical implementation of the radioligand.

## Methods

### Radiolabeling [^177^Lu]Lu-NeoB and [^175^Lu]Lu-NeoB

For each experiment performed in this study, NeoB (Advanced Accelerator Applications) was labeled with lutetium-177 (LuMark, IDB Holland) with a molar activity of 100 MBq/nmol. For the toxicity experiments, NeoB was also labeled with lutetium-175. For all experiments with [^177^Lu]Lu-NeoB, the radiochemical yield was > 90% and the radiochemical purity was > 88%. For all experiments with [^175^Lu]Lu-NeoB, the chemical yield was > 99%. A more detailed description of the radiolabeling can be found in the Supplementary Information.

### Animals

Male and female Balb/c AnNRj mice (Janvier) were maintained in standard individually ventilated cages (800 cm^2^, 8–10 animals per cage) with a 12-h light/dark cycle. Animals received water and food (SDS rat and Mouse Breeder and Grower (CRM[P])) ad libitum. On arrival, mice were left to acclimate for a minimum of 1 week and animals were 6–7 weeks old at the start of the experiments. All animals received a subcutaneous ID chip (UNO bv) on the left flank under isoflurane anesthesia. All conducted animal experiments were approved by the Erasmus MC Animal Welfare Committee and were in accordance with European law.

### Administration of [^177^Lu]Lu-NeoB

Both the biodistribution and the radiotoxicity of [^177^Lu]Lu-NeoB were studied after administration of 3 different dosages of the radioligand: 40 MBq/400 pmol [^177^Lu]Lu-NeoB, 80 MBq/800 pmol [^177^Lu]Lu-NeoB, and 120 MBq/1200 pmol [^177^Lu]Lu-NeoB. The different dosages were acquired by diluting the stock [^177^Lu]Lu-NeoB solution with PBS + kolliphor HS 15 (1 mg/mL). [^177^Lu]Lu-NeoB was administered intravenously (tail) to conscious animals in a total volume of 200 µL.

### Biodistribution study

Each dosage of [^177^Lu]Lu-NeoB was injected in *N* = 15 healthy male animals. At 5 different time points post injection (p.i.) (*t* = 1, 4, 24, 48, and 96 h), 3 animals per dosage group were sacrificed, after which blood and organs were collected and measured to determine radioactivity uptake.

Since in the radiotoxicity studies animals received 3 injections of the radioligand at 1-week intervals, an additional group of 3 male animals were injected twice with 120 MBq/1200 pmol [^177^Lu]Lu-NeoB at a 7 days (168 h) interval. This was followed by a biodistribution at t = 4 h p.i. of the second injection in order to investigate whether a previous injection affects the biodistribution of a subsequent injection administered after a seven day interval. In addition, to determine the amount of radioactivity remaining in the blood and organs right before a second injection, 3 additional male animals were injected with 40 MBq/400 pmol [^177^Lu]Lu-NeoB and the biodistribution was determined at *t* = 168 h. Moreover, to determine whether the biodistribution differs between male and female animals, 15 healthy female animals were injected with 120 MBq/1200 pmol [^177^Lu]Lu-NeoB and sacrificed at *t* = 1, 4, 24, 48, and 96 h p.i. A schematic overview of the biodistribution study can be found in the Supplemental Information (Fig. S1).

In the biodistribution study, the adrenal glands, brain, intestines, heart, kidneys, liver, lungs, mammary fat pad, pancreas, muscle, spleen, stomach, prostate gland (M), testes (M), ovaries (F), and urinary bladder were collected, weighed, and measured for radioactivity uptake using a gamma counter (1480 WIZARD automatic γ counter; PerkinElmer). To calculate the percentage injected activity (%IA), a vial containing 200 µL of a 1:1000 dilution of the injected [^177^Lu]Lu-NeoB solutions was also measured in the gamma counter. The %IA of all organs and tumor was corrected for the percentage of radioactivity left measured in the tail (injection site).

### Toxicity study

**Fig. 1:**
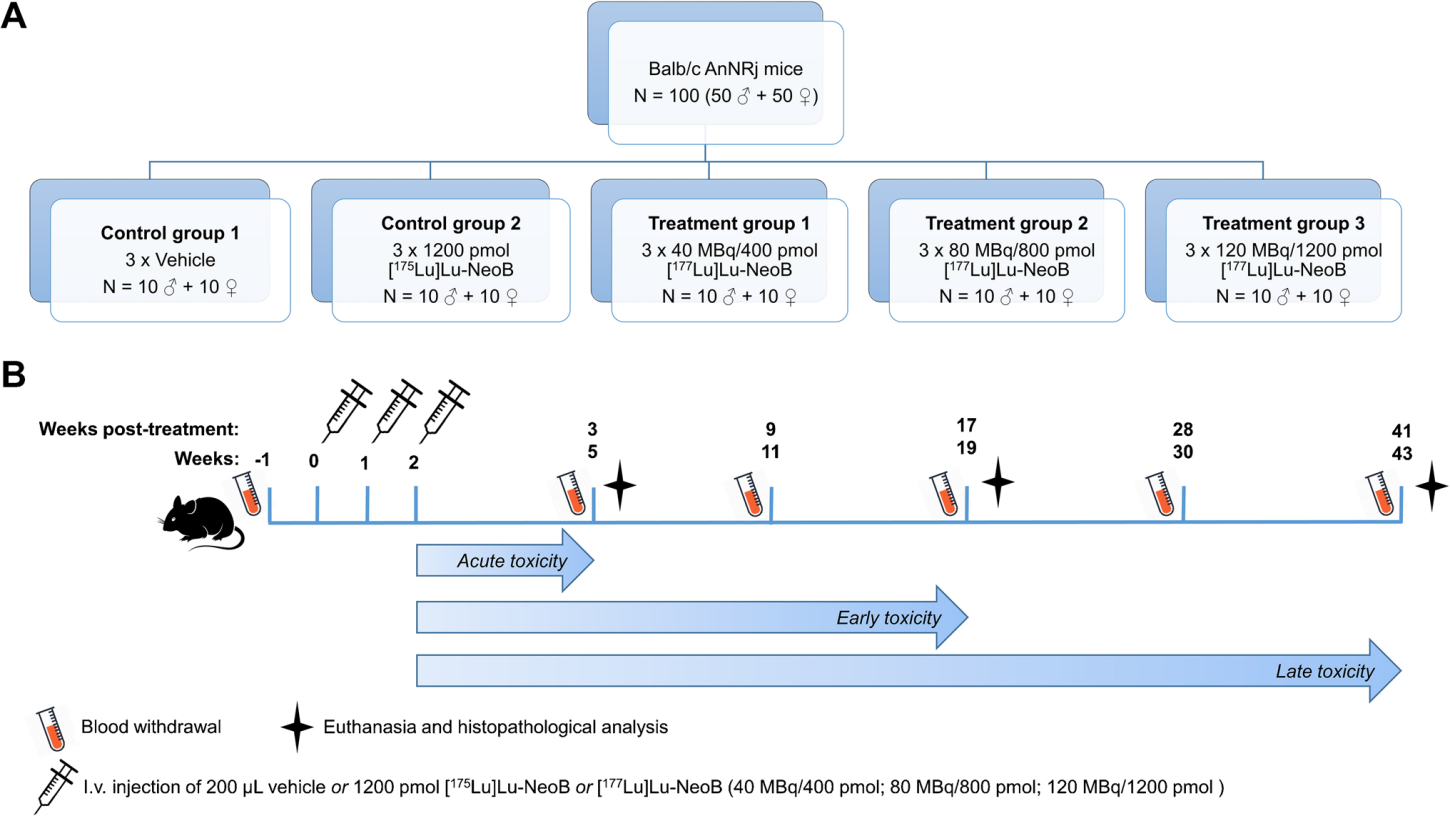
**A** Schematic overview of the 5 study groups of the toxicity study and **B** the time schedule of injections, blood withdrawal and animal sacrifice. i.v. = intravenous

Figure 1 shows a schematic overview of the toxicity study. Five different experimental groups (10 male and 10 female healthy animals per group; *n* = 100) were included in the radiotoxicity study and treated as follows: control group 1: 3 × vehicle (PBS + kolliphor HS 15 (1 mg/mL)); control group 2: 3 × 1200 pmol [^175^Lu]Lu-NeoB; treatment group 1: 3 × 40 MBq/400 pmol [^177^Lu]Lu-NeoB; treatment group 2: 3 × 80 MBq/800 pmol [^177^Lu]Lu-NeoB; and treatment group 3: 3 × 120 MBq/1200 pmol [^177^Lu]Lu-NeoB.

Animals received 3 injections with an interval of 7 days between each injection.

At 5, 19, and 43 weeks after the first injection, 3–4 male and 3–4 female animals of each group were sacrificed by isoflurane overdose after a blood sample was taken by retro-orbital puncture. Hereafter, the axillary lymph nodes, mesenteric lymph nodes, skin, mammary fat pad, sternum, pancreas, adrenal glands, kidneys, spleen, liver, gallbladder, stomach, duodenum, jejunum, ileum, caecum, colon, rectum, testes (M), epididymis (M), seminal vesicles (M), prostate gland (M), ovaries (F), oviducts (F), uterus-cervix (F), vagina (F), bladder, ureters, sciatic nerve, skeletal muscle, femur, lungs, trachea, esophagus, thymus, thyroid gland, aorta, heart, brain, pituitary gland, eyes, harderian glands, tongue, spinal column, and the tail were collected and analyzed macroscopically. All organs and tissues were formalin fixed (4% buffered formaldehyde) and shipped to the European Research Biology Centre (ERBC, Pomezia, Rome, Italy) where histological sampling, embedding, sectioning (5 µm thick), and hematoxylin–eosin staining were performed according to standard guidelines. All organs and tissues were analyzed microscopically by an experienced pathologist. A 5-point grading scale was used: 1: minimal change, 2: mild change, 3: moderate change, 4: marked change, 5: severe change.

Throughout the study, the animals were observed daily to check for physical signs of toxicity and signs of discomfort, and animals were weighed 2–3 times per week. Furthermore, at week -1, 5, 11, 19, 30, and 43, blood samples were collected by submandibular puncture from all animals that were in the study at that time point. All blood samples were analyzed for hematological parameters (hematocrit; hemoglobin; red blood cell count; white blood cell count; hemoglobin; mean cell hemoglobin concentration; mean cell volume; platelet count) on the scilVet abc + counter (Covetrus).

In addition, the following clinical chemistry parameters were measured: sodium, potassium chloride, calcium, glucose, creatinine, urea, alanine aminotransferase (ALT), aspartate aminotransferase (AST), alkaline phosphatase (ALP), amylase, lipase, and cholesterol, using the Roche/Hitachi cobas c systems according to the standard protocol. To reach the volume necessary for measuring the clinical chemistry parameters, the blood plasma was diluted 3–5 times with demineralized water.

### Dosimetry

Data obtained during the in vivo biodistribution studies were used to calculate the absorbed dose for each organ according to the MIRD-scheme [16]. The time integrated activity concentration coefficients were determined by fitting exponential curves to the activity concentration data points at 1, 4, 24, 48, 96, and 168 h p.i. and integrating these curves over time. The least squares fitting analysis was performed with GraphPad Prism software. The S-values for lutetium-177 were based on the 24 g realistic RADAR mouse phantom [17, 18]. The standard organ weights of this model were used to convert from activity concentration to activity. With this model, the absorbed dose in most organs could be calculated. S-values for organs not included in the mouse model, such as adrenal glands and prostate, were determined with the spherical node model within the Olinda/EXM code [19].

### Statistics

Statistical analysis was performed using GraphPad PRISM software (version 5). Significance levels were set at 5%. A simple binary logistic regression analysis was performed to determine a dose–effect relation between the absorbed dose and the histopathological findings in the kidneys using the log-likelihood ratio test as a significance indicator. The weight gain over time of control group 2 and treatment groups 1, 2, and 3 was compared to control group 1 by performing a two-way multiple comparison analysis of variance (ANOVA) with a Bonferroni correction. Significant outliers in the blood values set were detected with the Grubbs’ test and excluded from the data set. At a given time point, the measurements of all study groups were compared by performing a one-way ANOVA and in case of significance a Tukey test to correct for multiple comparisons. All data are represented as mean ± standard deviation (SD).

## Results

### Biodistribution study

#### Organ uptake

**Fig. 2:**
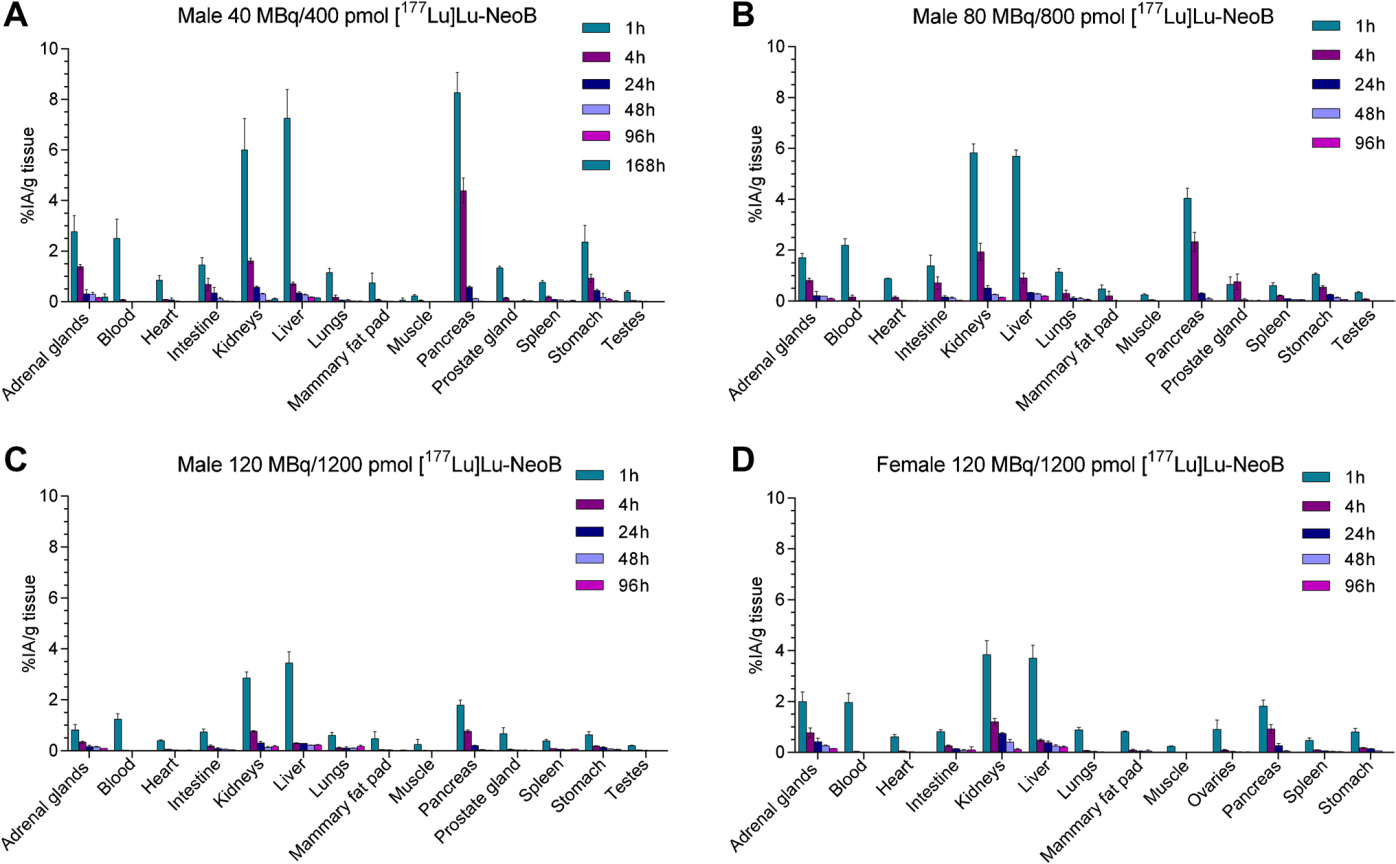
Biodistribution of [^177^Lu]Lu-NeoB in Balb/c mice. Uptake expressed as percentage of injected activity per gram of tissue (%IA/g) in male mice injected with **A** 40 MBq/400 pmol [^177^Lu]Lu-NeoB, **B** 80 MBq/800 pmol [^177^Lu]Lu-NeoB, **C** 120 MBq/1200 pmol [^177^Lu]Lu-NeoB, and **D** female mice injected with 120 MBq/1200 pmol [^177^Lu]Lu-NeoB. *N* = 3 in all cases except the 1 h time point of the male 40 MBq/400 pmol [^177^Lu]Lu-NeoB group (*n* = 2). Error bars represent the standard deviation

Figure 2 depicts the biodistribution results of male animals injected with 40 MBq/400 pmol [^177^Lu]Lu-NeoB (Fig. 2A), 80 MBq/800 pmol [^177^Lu]Lu-NeoB (Fig. 2B), 120 MBq/1200 pmol [^177^Lu]Lu-NeoB (Fig. 2C), and female animals injected with 120 MBq/1200 pmol [^177^Lu]Lu-NeoB (Fig. 2D). For all dosages, the highest organ uptake was observed at 1 h p.i., and for all groups, the highest uptake values were found in the kidneys, liver, and pancreas. Animals injected with the highest radioactive dose and peptide mass (120 MBq/1200 pmol [^177^Lu]Lu-NeoB) showed the overall lowest %IA/g for each tissue. For example, at 1 h p.i., pancreatic uptake was 1.79 ± 0.2%IA/g in male animals of the 120 MBq/1200 pmol group, while pancreatic uptake in the 80 MBq/800 pmol and the 40 MBq/400 pmol groups were 4.05 ± 0.40 and 8.27 ± 0.80%IA/g, respectively. However, while %IA/g was lowest for the 120 MBq/1200 pmol [^177^Lu]Lu-NeoB group, absolute radioactivity uptake in most organs was highest in this group compared to the other two groups. This was not the case for the pancreas, where the absolute radioactivity uptake was comparable between groups, despite the higher amount of radioactivity injected. The uptake values for each organ at all time points studied can be found in the Supplementary Information (Tables S3–S6). No significant differences in uptake and dose were found between male animals injected once, and male animals injected twice at a seven-day interval with 120 MBq/1200 pmol (Supplementary Fig. S2). Significant differences in uptake between male and female animals were observed for the adrenal glands and kidneys at 1 h, 4 h, 24 h, and 48 h p.i. Furthermore, 1 h and 4 h p.i. female mice showed a significantly higher uptake in the pancreas compared to male animals (Supplementary Fig. S3).

#### Dosimetry

**Fig. 3:**
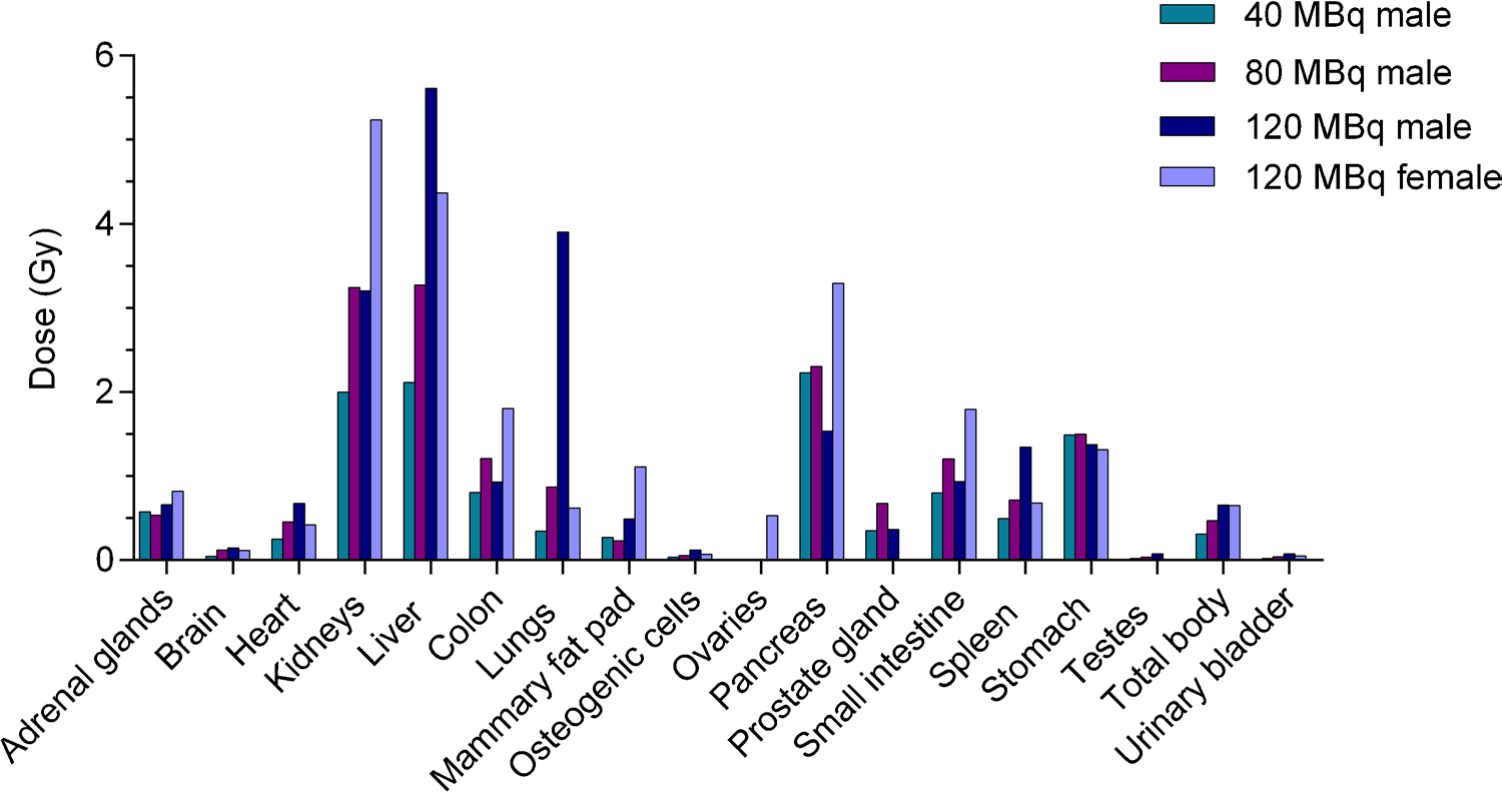
Absorbed organ doses of [^177^Lu]Lu-NeoB in Balb/c mice

Dosimetry calculations (Fig. 3, Table 1) revealed the highest absorbed doses in the pancreas, kidneys, and liver for all groups. The highest dose absorbed by the pancreas (3.3 Gy) was observed in female animals that received 120 MBq/1200 pmol [^177^Lu]Lu-NeoB. The male animals received a pancreatic dose of 2.2 Gy, 2.3 Gy, and 1.5 Gy for the 40 MBq/400 pmol, 80 MBq/800 pmol, and the 120 MBq/1200 pmol [^177^Lu]Lu-NeoB group, respectively. In addition, the female animals that received 120 MBq/1200 pmol [^177^Lu]Lu-NeoB also received the highest renal dose of 5.2 Gy (Table 1). The higher initial uptake in the female kidneys leads to a higher absorbed dose compared to the male kidneys, despite the shorter clearance half-life (Supplementary Fig. S4). The high absorbed dose in the lungs of male animals receiving 120 MBq/1200 pmol [^177^Lu]Lu-NeoB is due to the unexpectedly increased radioactivity uptake observed at the 96 h time point, which led to a high clearance half-life.

**Table 1:** Absorbed doses and extrapolated cumulative absorbed doses (Gy) in the main organs of mice injected with 1 × and 3 × [^177^Lu]Lu-NeoB, respectively

	40 MBq/400 pmol (M)		80 MBq/800 pmol (M)		120 MBq/1200 pmol (M)		120 MBq/1200 pmol (F)	
*Times injected:*	1 ×	3 ×	1 ×	3 ×	1 ×	3 ×	1 ×	3 ×
Adrenal glands	0.58	1.74	0.54	1.62	0.66	1.99	0.82	2.47
Brain	0.05	0.15	0.12	0.36	0.15	0.44	0.12	0.35
Heart wall	0.25	0.75	0.46	1.38	0.68	2.03	0.42	1.27
Kidneys	2.00	6.00	3.25	9.75	3.21	9.62	5.24	15.71
Liver	2.11	6.33	3.28	9.84	5.61	16.84	4.37	13.11
Colon wall	0.81	2.43	1.21	3.63	0.93	2.80	1.81	5.43
Lungs	0.35	1.05	0.87	2.61	3.91	11.72	0.62	1.87
Mammary fat pad	0.27	0.81	0.23	0.69	0.49	1.47	1.11	3.34
Osteogenic cells	0.03	0.09	0.06	0.18	0.12	0.37	0.07	0.21
Ovaries	-	-	-	-	-	-	0.53	1.60
Pancreas	2.23	6.69	2.31	6.93	1.54	4.61	3.30	9.89
Prostate gland	0.35	1.05	0.68	2.04	0.37	1.10	-	-
Small intestine	0.80	2.40	1.21	3.63	0.94	2.81	1.80	5.40
Spleen	0.50	1.50	0.72	2.16	1.35	4.05	0.68	2.05
Stomach wall	1.50	4.50	1.50	4.50	1.38	4.14	1.31	3.94
Testes	0.02	0.06	0.04	0.12	0.08	0.23	-	-
Total body	0.31	0.93	0.47	1.41	0.66	1.97	0.65	1.95
Urinary bladder	0.02	0.06	0.04	0.12	0.08	0.24	0.05	0.15

The absorbed doses determined from the biodistribution assay were extrapolated to the dose the animals received during the radiotoxicity study. Here, the animals received 3 consecutive injections with a 7-day interval. The cumulative doses from the toxicity study are presented in Table 1.

### Toxicity study

#### Tolerability

**Fig. 4:**
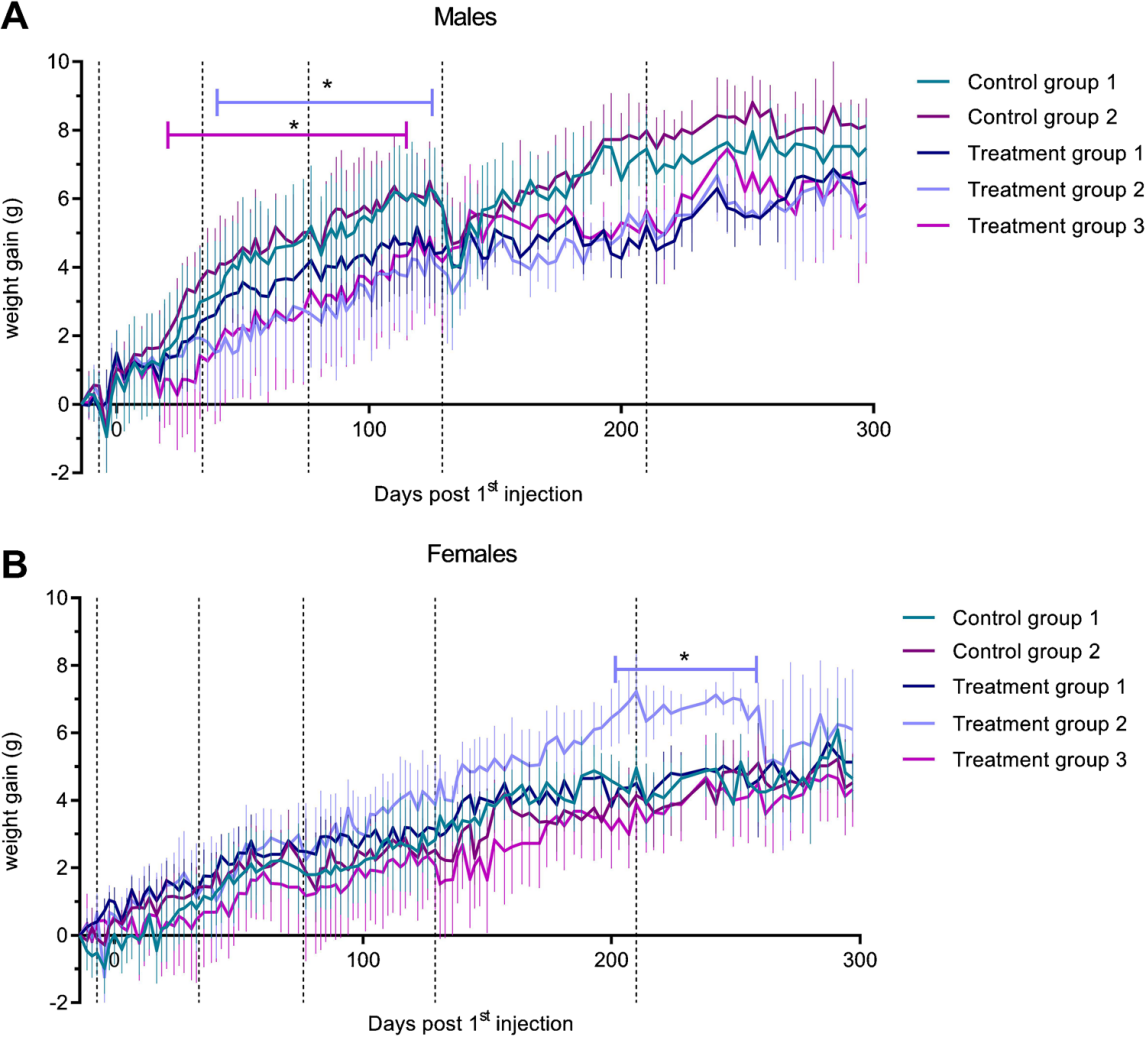
Weight of **A** male and **B** female animals during the toxicity study. Black dotted vertical lines indicate the time points of blood withdrawal. Error bars represent the standard deviation. *p < 0.05

Body weight was monitored to examine the general tolerability of the different dosages of [^177^Lu]Lu-NeoB administered (Fig. 4). The blood collection procedure resulted in weight decreases in all groups, but mice recovered quickly. The male treatment groups 2 and 3 showed a significantly less pronounced weight gain during the first half of the study compared to the control groups. At the end, the divergence between the male control groups and the treatment groups decreased. Female treatment group 2 showed significantly higher weight gain by the end of the study compared to control group 1; however, weight loss was observed during the last weeks of the study.

#### Histopathology

At three different time points after injection, histological examination was performed on sections of excised organs to determine histopathological changes related to acute, early, or late toxicity (Supplementary Tables S7–S12). At the first time point of sacrifice (week 5), minimal or mild treatment-related changes in the bladder were observed microscopically in the form of cytoplasmic vacuolation of urothelial cells of the urinary bladder epithelium for male treatment group 3 (3/3) and female treatment groups 2 (3/3) and 3 (3/3). For all mice, this observation was associated with mononuclear inflammatory cell infiltrates in the submucosa. This finding was also detected at week 19, mainly for male treatment group 2 (3/3) and all female treatment groups (7/9).

At the second time point of sacrifice (week 19), which was used to assess early toxicity, changes were observed in the kidneys, mainly in the form of hydronephrosis. Hydronephrosis was characterized by marked dilation of the renal pelvis and calyx, and was associated with severe cortical atrophy. This finding was present, mostly bilateral, in male treatment group 2 (3/3) and 3 (2/3), and female treatment group 1 (1/3) and 3 (3/3). Mild nephropathy (i.e., degenerative changes in the renal nephrons) was also noted in some cases. Ureteral dilatation was observed in correlation with hydronephrosis, when ureters were present in the histological sections.

At the final time point of sacrifice (week 43), minimal histopathological findings in the bladder were still noted in a few cases in male treatment group 2 and female treatment group 1 and 2. Marked or severe hydronephrosis with associated occasional mild nephropathy and ureteral dilation was detectable in the kidneys of male treatment groups 2 (2/4) and 3 (3/4) and female treatment groups 2 (1/4) and 3 (2/4). In addition, marked ovarian atrophy, in the absence of ovarian follicles and *corpora lutea*, with relatively elevated stroma was present in almost all treated females (10/11). Uterine and vaginal atrophy was also reported for the all but one affected female animal.

**Fig. 5:**
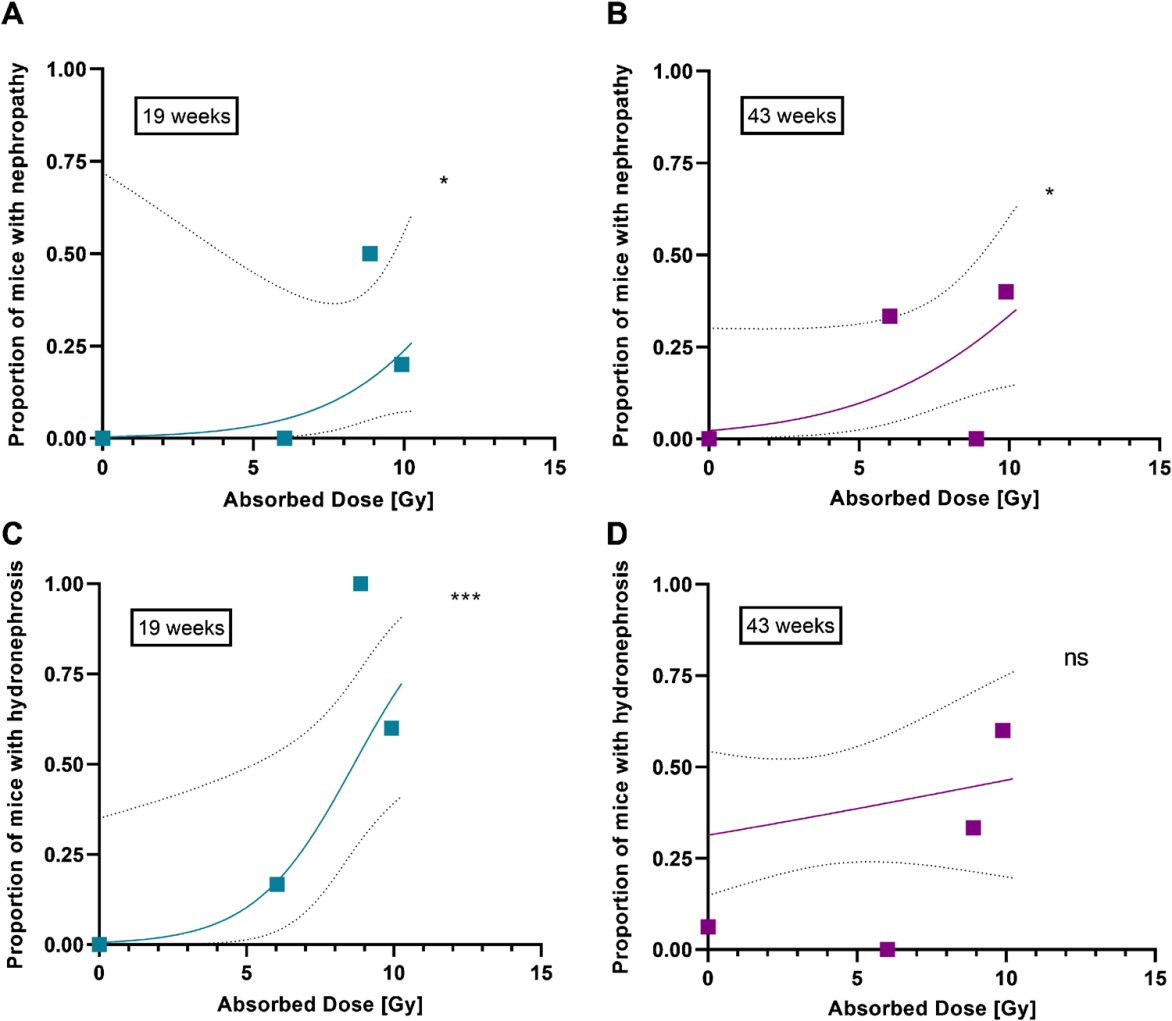
Dose–effect curves for histologically determined (**A** and **B**) nephropathy and (**C** and **D**) hydronephrosis at week 19 (left; *n* = 30) and week 43 (right; *n* = 38) in mouse kidneys (males and females combined). ns = not significant, *p < 0.05, ***p < 0.0001 based on the likelihood ratio test

Binary logistic regression analysis was performed to determine a dose–effect relationship for the incidence of renal toxicity (Fig. 5). A significant (*P* < 0.05) dose–effect was observed for nephropathy at both the early and late time points. For hydronephrosis, the strongest dose–effect relation was seen at 19 weeks (*P* < 0.0001) with an ED50 of 8.65 Gy (95% CI: 6.66–11.18 Gy).

#### Hematology

**Fig. 6:**
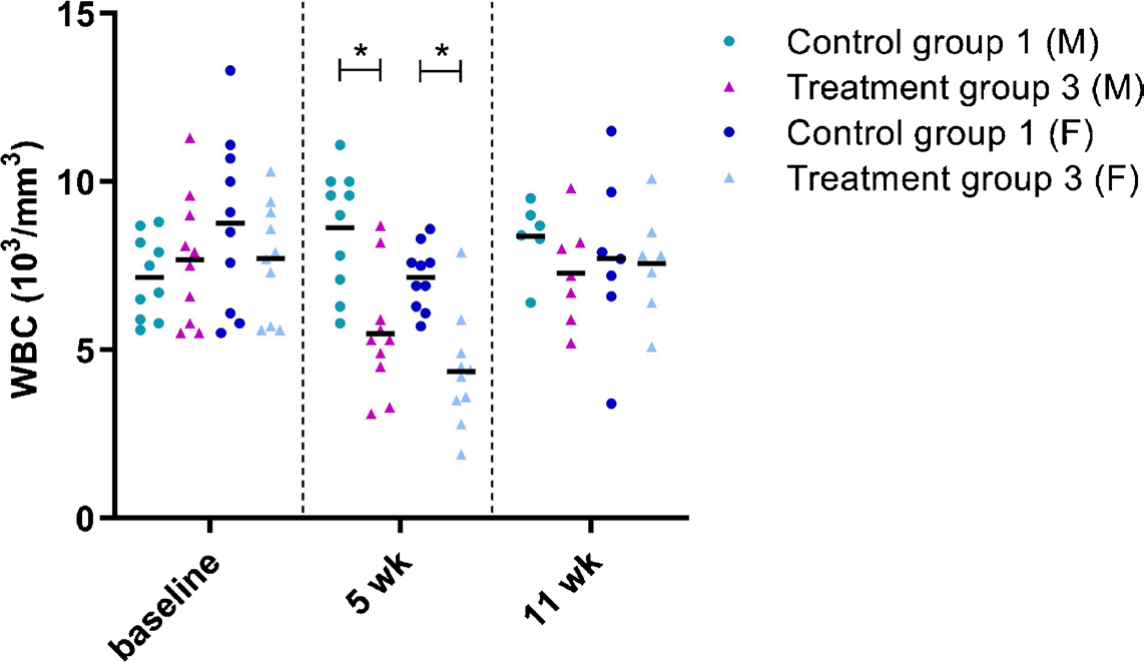
White blood cell (WBC) count in male (M) and female (F) control group 1 and treatment group 3 at baseline, week (wk) 5, and wk 11. Each dot represents an individual mouse. The black line depicts the group mean. *p < 0.05

The first series of blood analyses evaluated the hematologic profile (Supplementary Tables S13–S16). A treatment-related low white blood cell (WBC) count was observed at week 5 for treatment group 3 compared to control group 1 for both sexes (− 36.5% for males and − 39% for females vs control group 1). However, this was temporary as no significant differences in WBC counts were found at later time points (Fig. 6). Moreover, at week 30, a slight increase in WBC was seen in high dose males (+ 19.4% vs. control group 1).

All other differences in hematologic parameters compared to control data, including statistically significant differences, were unrelated to treatment and without a clear dose relationship. The values of these parameters were sporadic, consistent with normal biological variation, and/or negligible in magnitude.

#### Clinical chemistry

**Fig. 7:**
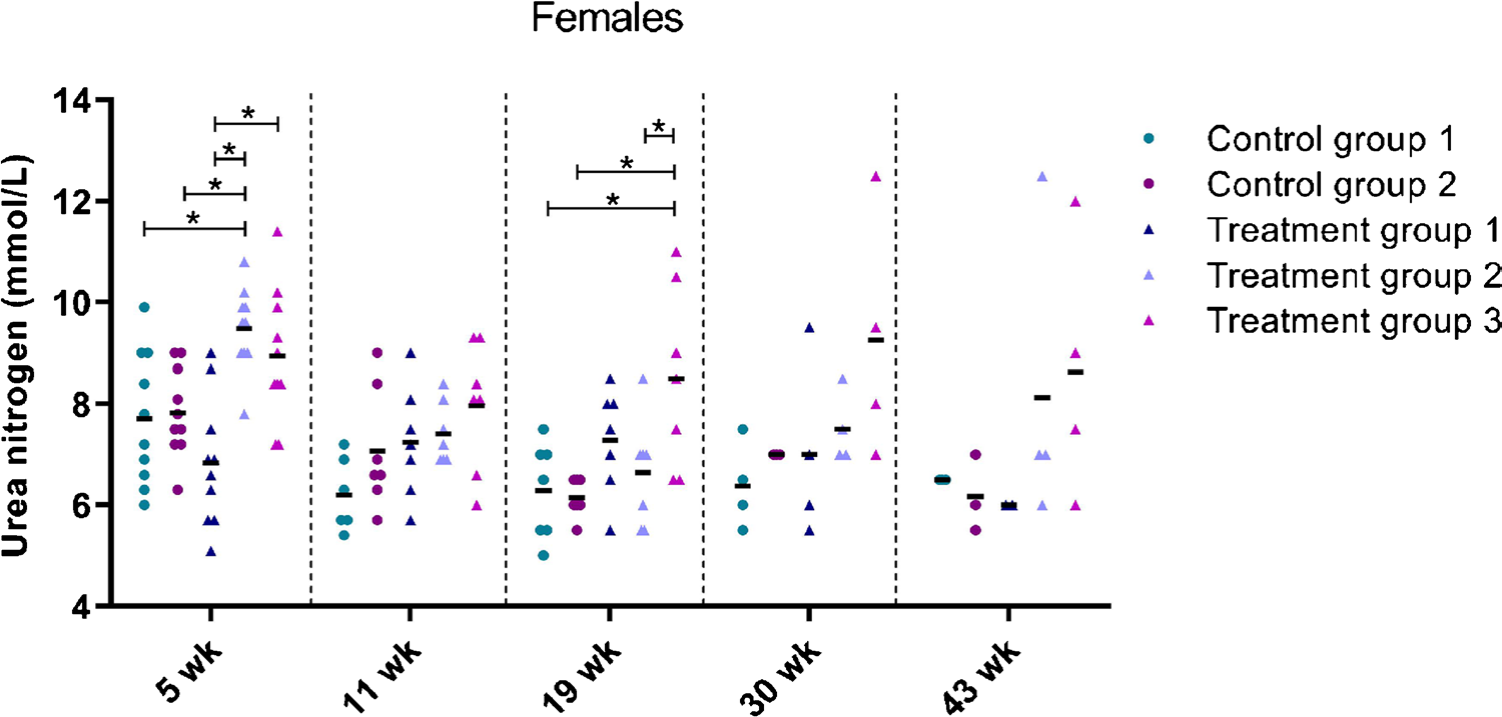
Blood urea nitrogen levels in female mice over time. Each dot represents an individual mouse. The black line depicts the group mean. wk = week, *p < 0.05

The second set of blood analyses involved clinical chemistry (Supplementary Tables S17–S20). Abnormalities were detected in blood urea nitrogen levels. These abnormalities were more pronounced in the female treatment groups than in the male treatment groups. A slight to moderate increase in mean urea values was seen in females of group 2 and 3 compared to control group 1 at weeks 5, 19, 30, and 43 (+ 20% and + 25% respectively at week 43). This observation showed to be dose related (Fig. 7).

A decrease in serum ALP concentration, which in rodents is usually related to liver and intestine function, was noted 3 weeks after treatment for male treatment group 3 compared to control group 1. Elevated ALP levels were observed at week 19 in male treatment group 2 and 3 compared to both control group 1 and 2. At week 43, a minimal increase in ALP was observed in individual males (maximum + 4.5% in group 3) and females (maximum + 8.7% in group 3).

No clear treatment-related abnormalities in blood levels were observed for ALT, AST, cholesterol, glucose, potassium, lipase, and amylase. Other statistically significant changes (sodium, chloride, calcium, and total protein) were not consistent and were therefore considered negligible.

## Discussion

Extensive preclinical safety evaluations are of great importance to support the conduct of clinical trials in humans. In this study, we characterized the toxicological profile of [^177^Lu]Lu-NeoB by evaluating acute, early, and late radiotoxicity in healthy mice following repeated administration of 40 MBq/400 pmol, 80 MBq/800 pmol, and 120 MBq/1200 pmol of the radioligand. By performing extensive biodistribution studies using the same dosages, we were able to correlate the radiation absorbed doses to the healthy organs with the observed toxicities.

Biodistribution studies revealed the liver, kidneys, and pancreas as the organs that received the highest absorbed doses for each tested dosage. These findings are in agreement with previously performed diagnostic and biodistribution studies where, besides the tumor, these organs showed the highest uptake of radiolabeled NeoB [11–13]. Furthermore, clinical diagnostic studies reported the kidneys and pancreas as possible dose-limiting organs, as they showed relatively high uptake [10, 13]. First in-human dosimetry investigations with another radiolabeled GRPR-antagonist, [^177^Lu]Lu-RM2, also identified the pancreas as a potential dose-limiting organ [20].

Organ toxicity was studied by histopathological examination of all organs and by measuring hematological and clinical chemistry blood parameters.

As mentioned above, the GRPR-expressing pancreas is often named as a potential dose-limiting organ for GRPR-targeting radionuclide therapy [12, 21, 22]. Pancreatitis can be diagnosed by an increase in serum lipase and amylase levels [23], neither of which were consistently found in this study. Furthermore, no histopathological signs of continuous or severe pancreatic toxicity was observed. These findings are in agreement with the study by Montemagno et al. (2021) who also reported no pancreatic alterations 100 days after mice bearing gastrointestinal stromal tumors were treated once weekly for 3 consecutive weeks with 37 MBq/400 pmol [^177^Lu]Lu-NeoB [24].

Previous preclinical and clinical studies with compounds for radioligand therapy (small molecules/peptides) have demonstrated that the kidneys (as the main elimination route) often receive higher radiation exposure than other organs. Similarly, as expected, the most significant radiotoxic effects detected during this study were found in the kidneys, namely in the kidneys of the animals administered with the highest dosage (male and female) at both the early and late time point. The absorbed kidney dose these animals received varied between 9 and 16 Gy, and renal abnormalities were predominantly observed in the form of severe hydronephrosis and mild nephropathy during histopathological examination.

Hydronephrosis is usually a result of blockage in the urinary tract, which consists of the kidneys, the bladder, the ureters, and the urethra. In mice that were treated with the highest dosage of the radiotracer, ureteral dilatation was observed, which correlated with the detected hydronephrosis. Minimal damage to the bladder, in the form of inflammation and vacuolation of cells, was also observed after treatment. Furthermore, a dose–effect relationship was determined between the kidney dose received and both hydronephrosis and nephropathy.

In turn, the obstruction of urine outflow and swelling of the kidneys may ultimately result in deterioration of function [25]. This is consistent with the fact that nephropathy was observed in most mice with hydronephrosis in our study. However, in some cases, only nephropathy was detected, presumably the acute cause of ionizing radiation [26]. This indicates that ionizing radiation itself is at least partly responsible for the observed nephropathy in mice with hydronephrosis.

These pathological findings were further underlined by an increase in blood urea nitrogen levels [27]. The increase in urea nitrogen levels was significant only in male treatment group 2 and in female treatment groups 2 and 3, again underlining a dose-dependent relationship.

The observed histological kidney abnormalities can provide an explanation for the irregular weight gain observed in our studies. The progression of kidney disease can lead to weight loss due to decreased appetite [28], while fluid buildup in the body can lead to abnormal weight gain. Female treatment group 2 showed weight gain earlier in the study, which could indicate that the females reached a more severe state of urinary tract dysfunction at an earlier time point compared to male animals. This could also be explained by the higher dose to which the kidneys of the female animals were exposed. Female animals receiving 40 MBq/400 pmol [^177^Lu]Lu-NeoB and 120 MBq/1200 pmol [^177^Lu]Lu-NeoB showed no significant weight gain, so the weight gain of females receiving 80 MBq/800 pmol [^177^Lu]Lu-NeoB could be a mass-specific observation.

The renal toxicity observed in this preclinical study does not directly imply severe nephrotoxicity if [^177^Lu]Lu-NeoB is applied clinically. Due to the very small size of murine kidneys and the long range of β^−^ particles, the murine kidneys receive a more homogeneous radiation dose compared to the much larger human kidneys. This is also true for the bladder for which only minimal to mild damage was observed in our study. Together with the aforementioned difference in size between the mouse and human bladder, this indicates that it is unlikely that bladder toxicity will occur in humans. These considerations are supported by clinical experience with another lutetium-177 labeled peptide, [^177^Lu]Lu-DOTATATE, which revealed a low incidence of kidney and urinary bladder radiotoxicity [29–31]. In the study by Bergsma et al. [29] it was estimated that patients received an average kidney dose of 20.1 ± 4.9 Gy, which led to (subacute) renal toxicity in only 1% of patients. In the study by Gupta et al. [31] the incidence was higher; minimal to mild nephrotoxicity was observed in 6/79 cases. In the latter study a very dose-intensive treatment regimen was applied with cumulative exposure for 15 days, while in traditional PRRT a cumulative exposure occurs over the course of 168 days (e.g., 4 cycles every 8 weeks).

Bone marrow toxicity is a concern when using targeted radionuclide therapy with, for example, lutetium-177 labeled peptides with a long circulation time [21, 32]. Here, we found a decrease in WBC counts 5 weeks after radioligand injection in treatment group 3, which received bone marrow doses between 0.20 and 0.36 Gy. Although not clear from our histopathological analysis, this finding could potentially indicate bone marrow suppression. However, it should also be noted that the brief drop in WBC counts may have (partly) been caused by the reported urinary bladder inflammation. The observed drop in WBC counts was transient and reversible, indicating that in case of bone marrow suppression it was only temporary and treatment did not cause permanent damage. Consistent with our findings, studies in humans have shown a low incidence (11%) of hematological toxicity after [^177^Lu]Lu-DOTA-octreotate PRRT, with patients receiving an average absorbed bone marrow dose of 2 Gy [21].

Even though animals of all groups received significantly high absorbed doses to the liver, and male animals of treatment group 3 received a high absorbed dose to the lungs, no clear microscopic evidence of toxicity was observed in these organs. The high absorbed dose to the lungs of this treatment group was caused by the relatively high radioligand uptake at 96 h p.i. The exact mechanism behind the unexpected higher lung uptake compared to early time points, which was only observed in male animals, remains unclear and warrants further investigations. In relation to the liver, slight and sporadic changes in ALP blood levels were detected, but these were considered unrelated to the radioligand.

Furthermore, the ovaries of 10/11 treated female mice showed signs of late toxicity, which is in line with the known radiosensitivity of the ovarian follicle [33]. In case [^177^Lu]Lu-NeoB is to be used for the treatment of women with child-bearing potential, the risk of an impact on ovaries should be considered.

Although we performed extensive blood analyzes in this study, there were some limitations to the applied methods. First, since several studies have shown that most clinical chemistry parameters are age-dependent and variability can be easily introduced by e.g. time of sample collection, sample handling and storage [27], it was decided to compare all parameters of the treated animals with the control group at the different time points and not with their own baseline measurement. Second, baseline measurements were taken when mice were relatively young, meaning that most of the reference values generated were not representative for adult mice and baseline comparisons would reveal age-dependent rather than treatment-related effects [34]. Moreover, platelet counts could not be assessed because the blood collection method used resulted in unreliable platelet counts. Also, the creatinine values could not be included because measurements were below the limit for accurate detection.

In summary, in this preclinical in vivo study, treatment with [^177^Lu]Lu-NeoB was generally tolerated in male and female mice receiving 3 different dosages of [^177^Lu]Lu-NeoB: 40 MBq/400 pmol, 80 MBq/800 pmol, and 120 MBq/1200 pmol, administered 3 times with 1 week intervals. Kidneys have shown to be the dose limiting organs as they show signs of dose-dependent histological and biochemical abnormalities at early and late time points. The kidneys are not GRPR-expressing organs; however, they are the main excretion route for this compound. No clear signs of toxicity were found in the liver and pancreas, despite the high absorbed doses observed. The (often dose-limiting) bone marrow also showed no clear evidence of toxicity.

These findings underline the potential of [^177^Lu]Lu-NeoB as a safe option for clinical therapy, as even the highest dosage used in this repeated dose-intensive study did not result in toxicity in the GRPR-expressing pancreas, or extensive hematological damage. Although renal toxicity was observed in this study with [^177^Lu]Lu-NeoB treatment, clinical studies using [^177^Lu]Lu-DOTATATE revealed limited renal toxicity in patients at therapeutically effective doses. This suggests that the renal effects observed in this study may be a consequence of the dose-intensive schedule and the more homogeneous radiation dose received by mouse kidneys. Thus, such an effect may not be dose-limiting when [^177^Lu]Lu-NeoB is applied clinically.

## Supplementary Information

Below is the link to the electronic supplementary material.

Supplementary file1 (PDF 1.08 MB)

## Declarations

### Data availability

The datasets generated during and/or analyzed during the current study are available from the corresponding author on reasonable request.
