# Supplementary material for: Safety of [177Lu]Lu-NeoB treatment: a preclinical study characterizing absorbed dose and acute, early, and late organ toxicity
Source: Eur J Nucl Med Mol Imaging. 2022 Aug 11;49(13):4440–51. doi: 10.1007/s00259-022-05926-2 (PMC9605926; doi:10.1007/s00259-022-05926-2)
Supplement: Supplementary file 1 — Supplementary file1 (PDF 1.08 MB) [file 259_2022_5926_MOESM1_ESM.pdf]

Supplementary Information

Article title: Safety of [^{177}Lu]Lu-NeoB treatment: a preclinical study characterizing absorbed dose and acute, early, and late organ toxicity
Journal name: European Journal of Nuclear Medicine and Molecular Imaging (EJNMMI)
Author names: Eline A.M. Ruigrok, Marjolein Verhoeven, Mark W. Konijnenberg, Erik de Blois, Corrina M.A. de Ridder, Debra C. Stuurman, Luisa Bertarione, Katia Rolfo, Marion de Jong and Simone U. Dalm
Corresponding author: Simone U. Dalm; Erasmus MC, University Medical Center Rotterdam, Department of Radiology and Nuclear Medicine, Rotterdam, The Netherlands; s.dalm@erasmusmc.nl

Methods

Radiolabeling [^{177}Lu]Lu-NeoB and [^{175}Lu]Lu-NeoB

For lutetium-177 labeling, three solutions were prepared prior to radiolabeling:
Solution 1: 0.250 mL gentisic acid (20 mg/mL) + 1.75 mL acetate buffer (0.5 M, pH 4.5-5.0).
Solution 2: ~1 mg NeoB + 5 mL kolliphor HS 15 (2 mg/mL).
Solution 3: 3 mL ascorbic acid (22.5 mg/mL) + 1 mL acetate buffer (1 M, pH 5).
First, 0.5 mL of lutetium-177 (50 GBq/mL) was added to 1 mL of solution 1. Subsequently ~66 µL of solution 2 was added, and the mixture was left in a 95°C heating block for 7 minutes. After cooling, 500 µL of solution 3 and 420 µL MilliQ and 50 µL of DTPA (4 mM) were added. The radiochemical yield and the radiochemical purity of [^{177}Lu]Lu-NeoB were measured to determine the quality of the labeling. The radiochemical yield was measured by instant thin-layer chromatography on silica gel (Varian) using a 1.0 M aqueous solution of ammonium acetate:methanol (40:60 V/V) as the mobile phase. The radiochemical purity of [^{177}Lu]Lu-NeoB was measured by high-pressure liquid chromatography (HPLC) using an Alliance HPLC system (Waters) containing the W2998 PDA Detector. UV absorbance was measured at 278 nm and a peptide XB-C18 column (3.6 µm, 150 x 4.6 mm) was used with a gradient profile of 0.1% formic acid (A) and acetonitrile (B) (Table S2). Radioactivity was monitored with a system holding a NaI detector, digital multichannel analyzer and dedicated software (MetorX BV) connected to the HPLC system.
For the lutetium-175 labeling , a 1:1 molar ratio of lutetium-175 (ICP-standard 1g/L, Merck) was added to a labeling mixture containing NeoB and sodium-acetate as buffer, final pH 4.0. The mixture was left in a 95°C heating block for 20 minutes. To determine the chemical yield, UHPLC (Acquity H-Class, waters) with a W2998 PDA detector including a HSS-C18 (1.8 µm, 2.1 x 50 mm) column with a gradient profile of 20 mM ammonium acetate (A) acid and methanol (B) (Table S1) was used.

Table S1. UHPLC-method

Time (min)	A (%)	B (%)
0.0-0.14	100	0
0.14-0.2	35	65
0.2-3.5	5	95
3.5-3.51	0	100
3.51-4.0	0	100
4.0-4.01	100	0
4.01-4.5	100	0

Table S2. HPLC-method

Time (min)	A (%)	B (%)
0-2	85	15
2-9	60	40
9-11	60	40
11-11.5	0	100
11.5-13.0	0	100
13.0-13.1	85	15
13.1-15.5	85	15

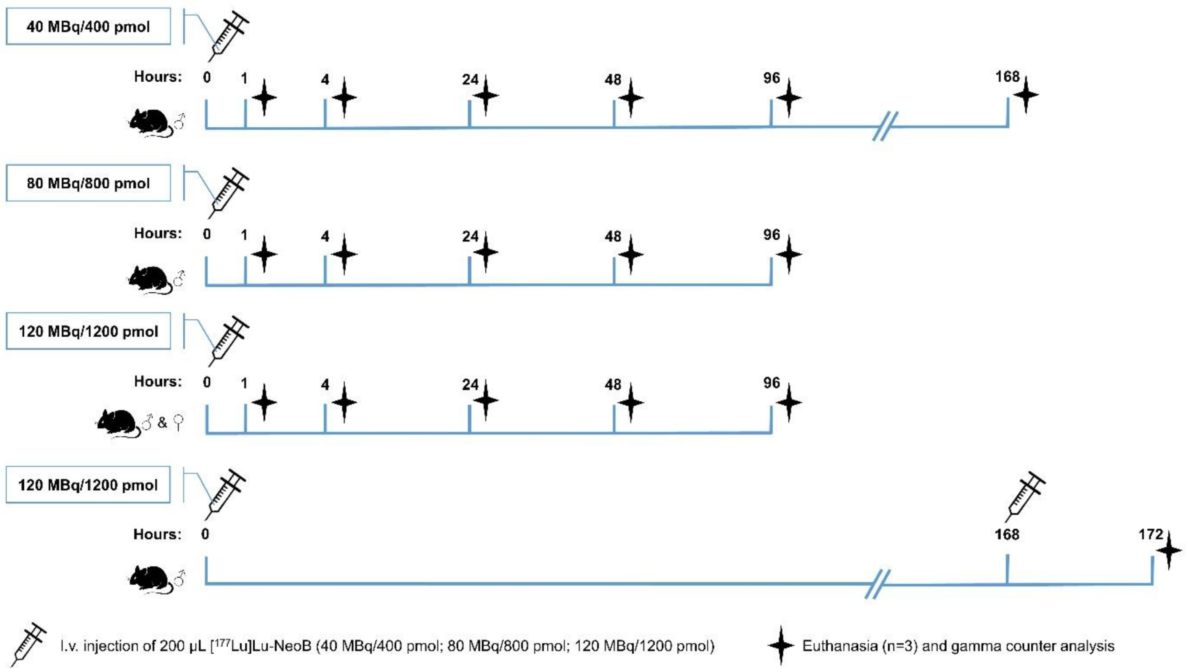

Figure S1: Schematic overview of the biodistribution study

Timelines of the biodistribution study per injected [^{177}Lu]Lu-NeoB dosage.

Table S3. Biodistribution uptake values (%IA/g) of all male animals injected with 40 MBq/400 pmol [^{177}Lu]Lu-NeoB.

	**1h***	**4h**	**24h**	**48h**	**96h**	**168h**
**Adrenal glands**	2.77±0.63	1.38±0.09	0.31±0.17	0.29±0.08	0.16±0.01	0.18±0.12
**Blood**	2.50±0.77	0.08±0.02	0.01±0.002	0.002±0.0004	0.001±0.0001	0.0005±0.0001
**Brain**	0.11±0.04	0.02±0.02	0.004±0.001	0.003±0.0003	0.001±0.0002	0.0015±0.001
**Heart**	0.85±0.19	0.09±0.01	0.07±0.07	0.02±0.003	0.008±0.003	0.01±0.0002
**Intestine**	1.45±0.29	0.68±0.24	0.35±0.21	0.12±0.05	0.03±0.01	0.01±0.004
**Kidneys**	6.01±1.24	1.62±0.11	0.57±0.05	0.30±0.03	0.06±0.01	0.11±0.02
**Liver**	7.25±1.14	0.71±0.07	0.34±0.05	0.27±0.02	0.19±0.01	0.14±0.01
**Lungs**	1.16±0.16	0.18±0.08	0.07±0.01	0.06±0.03	0.02±0.01	0.02±0.01
**Mammary fat pad**	0.74±0.39	0.08±0.02	0.03±0.01	0.02±0.001	0.01±0.002	0.06±0.0003
**Muscle**	0.23±0.05	0.05±0.02	0.01±0.003	0.005±0.001	0.002±0.001	0.002±0.0004
**Pancreas**	8.27±0.80	4.39±0.50	0.58±0.04	0.12±0.01	0.02±0.0004	0.01±0.001
**Prostate gland**	1.34±0.07	0.14±0.03	0.03±0.01	0.02±0.01	0.04±0.05	0.03±0.02
**Spleen**	0.76±0.07	0.20±0.03	0.09±0.004	0.07±0.01	0.02±0.003	0.05±0.01
**Stomach**	2.36±0.66	0.93±0.15	0.44±0.05	0.18±0.15	0.10±0.03	0.04±0.01
**Testes**	0.38±0.06	0.05±0.004	0.02±0.004	0.01±0.005	0.006±0.0003	0.01±0.0001^
**Urinary bladder**	3.19±0.81	2.43±1.79	0.17±0.14	0.08±0.04	0.04±0.03	0.02±0.004

***** Outlier excluded from dataset based on body weight (N = 2 for the 1h time point)

^ Testes could not be located for one mouse due to a shrunken reproductive system (N=2 at the 168 h time point)

Table S4. Biodistribution uptake values (%IA/g) of male animals injected with 80 MBq/800 pmol [^{177}Lu]Lu-NeoB.

	**1h**	**4h**	**24h**	**48h**	**96h**
**Adrenal glands**	1.70±0.17	0.81±0.09	0.21±0.17	0.20±0.001	0.10±0.01
**Blood**	2.19±0.26	0.16±0.08	0.005±0.001	0.002±0.0001	0.001±0.00001
**Brain**	0.11±0.01	0.07±0.10	0.004±0.001	0.003±0.001	0.002±0.0002
**Heart**	0.88±0.03	0.14±0.05	0.04±0.004	0.03±0.002	0.02±0.01
**Intestine**	1.39±0.43	0.71±0.24	0.16±0.06	0.12±0.04	0.04±0.01
**Kidneys**	5.83±0.35	1.93±0.34	0.51±0.10	0.25±0.02	0.14±0.02
**Liver**	5.70±0.24	0.91±0.19	0.34±0.01	0.28±0.02	0.19±0.02
**Lungs**	1.14±0.14	0.29±0.14	0.11±0.06	0.10±0.04	0.05±0.03
**Mammary fat pad**	0.48±0.15	0.20±0.18	0.02±0.01	0.02±0.004	0.01±0.002
**Muscle**	0.25±0.04	0.05±0.01	0.01±0.002	0.01±0.001	0.004±0.002
**Pancreas**	4.05±0.40	2.33±0.37	0.29±0.04	0.08±0.05	0.02±0.001
**Prostate gland**	0.66±0.30	0.76±0.31	0.05±0.04	0.02±0.01	0.02±0.02
**Spleen**	0.62±0.10	0.21±0.02	0.10±0.001	0.06±0.01	0.05±0.01
**Stomach**	1.05±0.05	0.55±0.07	0.25±0.02	0.13±0.02	0.06±0.01
**Testes**	0.34±0.03	0.08±0.01	0.02±0.004	0.01±0.001	0.01±0.002
**Urinary bladder**	16.25±23.63	6.97±5.71	0.12±0.03	0.09±0.05	0.03±0.004

Table S5. Biodistribution uptake values (%IA/g) of male animals injected with 120 MBq/1200 pmol [^{177}Lu]Lu-NeoB.

	**1h**	**4h**	**4h (168h after 1st injection)**	**24h**	**48h**	**96h**
**Adrenal glands**	0.82±0.21	0.34±0.05	0.34±0.11	0.16±0.06	0.15±0.02	0.10±0.003
**Blood**	1.24±0.21	0.02±0.001	0.04±0.02	0.004±0.0004	0.002±0.001	0.001±0.0002
**Brain**	0.08±0.04	0.01±0.003	0.01±0.001	0.004±0.002	0.004±0.003	0.003±0.001
**Heart**	0.40±0.03	0.05±0.005	0.06±0.01	0.03±0.004	0.02±0.01	0.02±0.004
**Intestine**	0.73±0.12	0.18±0.04	0.13±0.07	0.09±0.03	0.05±0.01	0.03±0.01
**Kidneys**	2.86±0.24	0.77±0.03	0.89±0.07	0.30±0.06	0.13±0.03	0.17±0.04
**Liver**	3.44±0.44	0.29±0.02	0.43±0.08	0.28±0.01	0.22±0.01	0.23±0.02
**Lungs**	0.61±0.11	0.11±0.03	0.10±0.01	0.11±0.05	0.10±0.01	0.17±0.05
**Mammary fat pad**	0.47±0.27	0.03±0.01	0.08±0.004	0.03±0.01	0.01±0.001	0.02±0.01
**Muscle**	0.24±0.20	0.02±0.003	0.03±0.02	0.01±0.001	0.004±0.001	0.004±0.001
**Pancreas**	1.79±0.20	0.76±0.06	0.88±0.13	0.20±0.02	0.04±0.002	0.02±0.004
**Prostate gland**	0.67±0.23	0.05±0.01	0.20±0.03	0.03±0.003	0.02±0.01	0.01±0.004
**Spleen**	0.39±0.06	0.09±0.002	0.10±0.01	0.05±0.004	0.04±0.002	0.07±0.002
**Stomach**	0.62±0.13	0.18±0.02	0.18±0.004	0.13±0.02	0.07±0.01	0.05±0.004
**Testes**	0.20±0.03	0.03±0.001	0.03±0.01	0.01±0.003	0.01±0.002	0.01±0.001
**Urinary bladder**	5.93±7.80	2.40±3.52	0.54±0.58	0.11±0.022	0.07±0.03	0.06±0.02

Table S6. Biodistribution uptake values (%IA/g) of female animals injected with 120 MBq/1200 pmol [^{177}Lu]Lu-NeoB.

	**1h**	**4h**	**24h**	**48h**	**96h**
**Adrenal glands**	2.00±0.38	0.77±0.19	0.41±0.14	0.25±0.04	0.14±0.02
**Blood**	1.96±0.36	0.04±0.01	0.01±0.002	0.003±0.001	0.001±0.0002
**Brain**	0.27±0.22	0.01±0.001	0.003±0.001	0.002±0.001	0.001±0.001
**Heart**	0.61±0.09	0.06±0.004	0.03±0.01	0.01±0.002	0.01±0.001
**Intestine**	0.81±0.07	0.26±0.03	0.14±0.01	0.08±0.03	0.10±0.12^
**Kidneys**	3.84±0.56	1.21±0.13	0.74±0.04	0.40±0.12	0.11±0.04
**Liver**	3.71±0.51	0.48±0.05	0.38±0.08	0.25±0.05	0.21±0.03
**Lungs**	0.88±0.09	0.07±0.02	0.03±0.01	0.02±0.01	0.01±0.005
**Mammary fat pad**	0.81±0.03	0.09±0.03	0.05±0.01	0.05±0.04	0.02±0.003
**Muscle**	0.24±0.02	0.02±0.002	0.01±0.001	0.004±0.002	0.002±0.001
**ovaries**	0.90±0.36	0.09±0.02	0.04±0.01	0.02±0.01	0.02±0.01
**Pancreas**	1.82±0.23	0.92±0.18	0.26±0.09	0.05±0.02	0.01±0.001
**Spleen**	0.47±0.09	0.10±0.01	0.06±0.004	0.04±0.004	0.03±0.01
**Stomach**	0.80±0.14	0.18±0.01	0.14±0.02	0.06±0.01	0.02±0.003
**Urinary bladder**	2.67±1.96	0.18±0.02	0.13±0.08	0.11±0.04	0.06±0.03

^ Large SD caused by deviating organ weight of one mouse

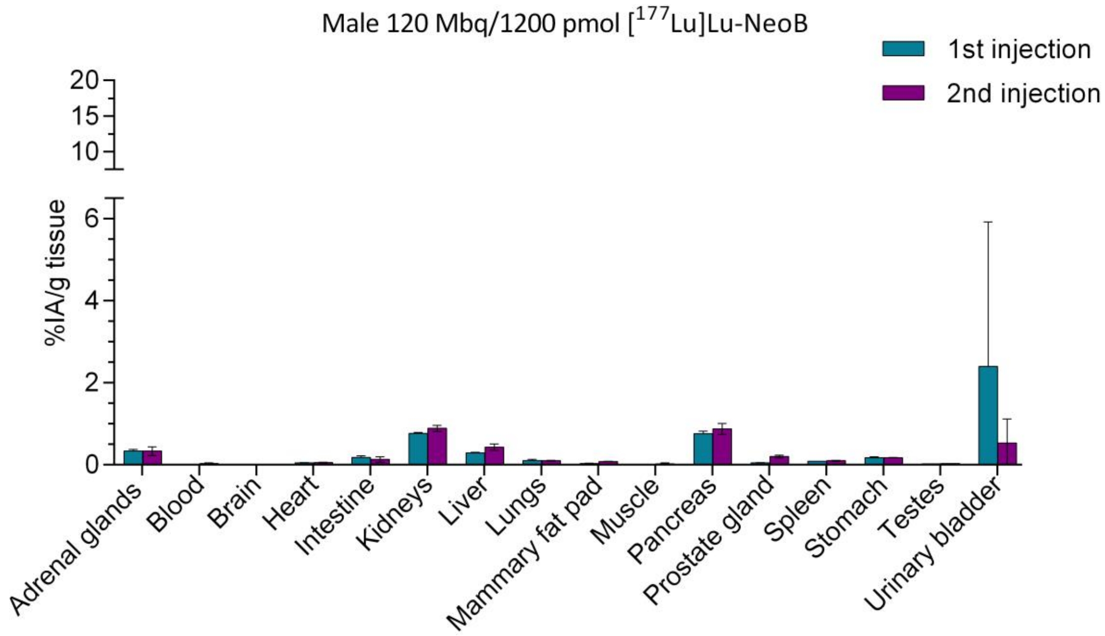

Figure S2: Biodistribution uptake values

Uptake expressed in percentage of injected activity per grams of tissue (%IA/g). Comparison in uptake between animals receiving one versus 2 injections with a 7-day interval.

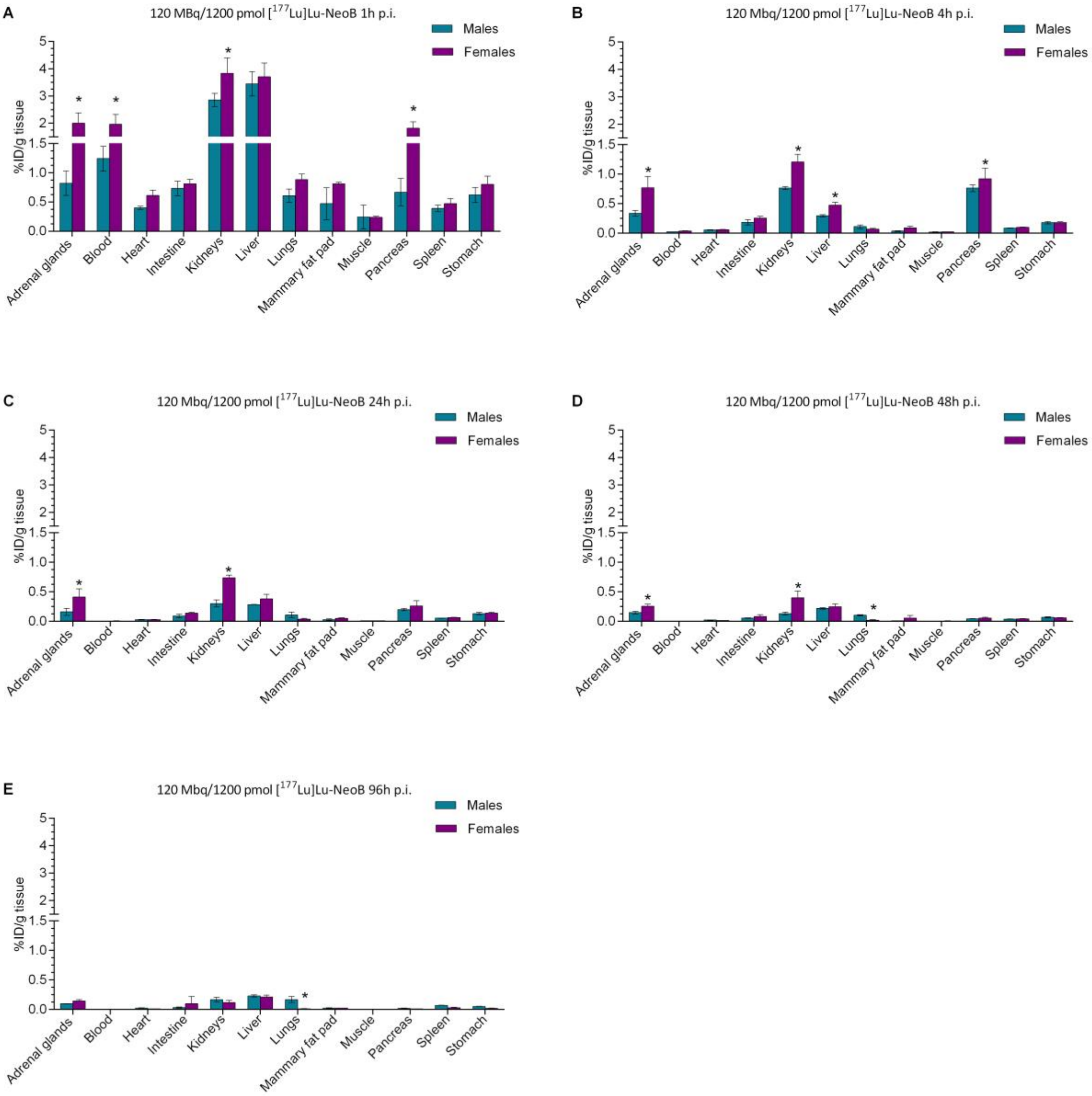

Figure S3: Biodistribution uptake values

Uptake expressed in percentage of injected activity per grams of tissue (%IA/g). Comparison in uptake between male and female animals receiving 120 MBq/1200 pmol [^{177}Lu]Lu-NeoB at (A) 1h, (B) 4h, (C) 24h, (D) 48h and (E) 96h after injection. Error bars represent the standard deviation.

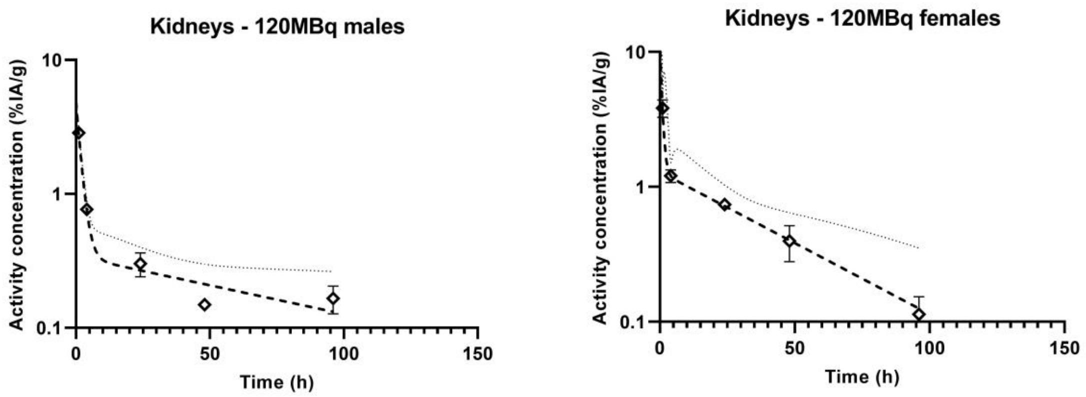

Figure S4: Pharmacokinetic modeling of [^{177}Lu]Lu-NeoB kidney clearance
Comparison in kidney clearance between male (left) and female (right) animals receiving 120 MBq/1200 pmol [^{177}Lu]Lu-NeoB. The exponential fit was performed using a two-phase decay model with an R^2 of 0.95 for males and 0.97 for females.

Table S7. Microscopic observations during histopathological evaluation of organs/tissues of male mice sacrificed 5 weeks after the first injection. A 5-point grading scale was used: 1. Minimal change, 2. Mild change, 3. Moderate change, 4. Marked change, 5. Severe change.

Sex	**Males**																								
Group	Control group 1					Control group 2					Treatment group 1					Treatment group 2					Treatment group 3				
N	3					3					3					3					3				
Score	1	2	3	4	5	1	2	3	4	5	1	2	3	4	5	1	2	3	4	5	1	2	3	4	5
Adrenal glands – Subcapsular cell hyperplasia		1/3				1/3	1/3				1/3														
Adrenal glands – accessory cortical tissue						1/3																			
Urinary bladder – inflammatory cell foci																					3/3				
Urinary bladder – urothelium vacuolation																					2/3	1/3			
Urinary bladder – mineralization																						1/3			
Testes – germ cell depletion												1/3				1/3					1/3				
Thyroid gland – ectopic thymus											1/3					1/3									
Spleen – extramedullary haematopoiesis							1/3					2/3													
Pancreas – acinar cell degeneration											1/3														
Pancreas – mineralization																						1/3			
Prostate gland – inflammatory cell foci																					2/3				
Heart – inflammatory cell foci																						1/3			

Table S8. Microscopic observations during histopathological evaluation of organs/tissues of female mice sacrificed 5 weeks after the first injection. A 5-point grading scale was used: 1. Minimal change, 2. Mild change, 3. Moderate change, 4. Marked change, 5. Severe change.

Sex	**Females**																								
Group	Control group 1					Control group 2					Treatment group 1					Treatment group 2					Treatment group 3				
N	3					3					3					3					3				
Score	1	2	3	4	5	1	2	3	4	5	1	2	3	4	5	1	2	3	4	5	1	2	3	4	5
Adrenal glands – Subcapsular cell hyperplasia	1/3					2/3					1/3	1/3				3/3					1/3				
Urinary bladder – inflammatory cell foci											1/3					1/3	2/3				1/3	2/3			
Urinary bladder – urothelium vacuolation																2/3	1/3				1/3	2/3			
Spleen – extramedullary haematopoiesis												2/3										1/3			
Thyroid gland – follicular cyst/s																					1/3				
Liver – inflammatory cell foci	1/3															1/3					1/3				
Brain - artifacts						1/3															1/3				

Table S9. Microscopic observations during histopathological evaluation of organs/tissues of male mice sacrificed 19 weeks after the first injection. A 5-point grading scale was used: 1. Minimal change, 2. Mild change, 3. Moderate change, 4. Marked change, 5. Severe change.

Sex	**Males**																								
Group	Control group 1					Control group 2					Treatment group 1					Treatment group 2					Treatment group 3				
N	3					3					3					3					3				
Score	1	2	3	4	5	1	2	3	4	5	1	2	3	4	5	1	2	3	4	5	1	2	3	4	5
Adrenal glands – Subcapsular cell hyperplasia	2/3					2/3					1/3	1/3				1/3	1/3				2/3	1/3			
Adrenal glands – accessory cortical tissue	1/3																								
Urinary bladder – inflammatory cell foci																2/3									
Urinary bladder – urothelium vacuolation																3/3					1/3				
Urinary bladder – proteinaceous plug		1/3					1/3																		
Testes – germ cell depletion											2/3														
Thyroid gland – ectopic thymus											1/3										1/3				
Spleen – extramedullary haematopoiesis												1/3										1/3			
Pancreas – inflammatory cell foci		3/3				1/3											2/3								
Kidneys – hydronephrosis																		1/3	2/3					2/3	
Kidneys – nephropathy																		1/3					1/3		

Table S10. Microscopic observations during histopathological evaluation of organs/tissues of female mice sacrificed 19 weeks after the first injection. A 5-point grading scale was used: 1. Minimal change, 2. Mild change, 3. Moderate change, 4. Marked change, 5. Severe change.

Sex	**Females**																								
Group	Control group 1					Control group 2					Treatment group 1					Treatment group 2					Treatment group 3				
N	3					3					3					3					3				
Score	1	2	3	4	5	1	2	3	4	5	1	2	3	4	5	1	2	3	4	5	1	2	3	4	5
Adrenal glands – Subcapsular cell hyperplasia	2/3	1/3				1/3	2/3				1/3	2/3				2/3	1/3				1/3	2/3			
Urinary bladder – inflammatory cell foci											2/3	1/3				2/3					1/3	1/3	1/3		
Urinary bladder – urothelium vacuolation											2/3					2/3	1/3				1/3	1/3			
Spleen – extramedullary haematopoiesis																	1/3								
Pancreas – inflammatory cell foci	1/3										2/3					1/3					1/3	1/3			
Kidneys – hydronephrosis														1/3										3/3	
Kidneys – nephropathy																						1/3			
Liver – inflammatory cell foci	1/3										1/3	1/3				2/3									
Ureter(s) - dilatation																						1/3			

Table S11. Microscopic observations during histopathological evaluation of organs/tissues of male mice sacrificed 43 weeks after the first injection. A 5-point grading scale was used: 1. Minimal change, 2. Mild change, 3. Moderate change, 4. Marked change, 5. Severe change.

Sex	**Males**																								
Group	Control group 1					Control group 2					Treatment group 1					Treatment group 2					Treatment group 3				
N	4					4					3					4					4				
Score	1	2	3	4	5	1	2	3	4	5	1	2	3	4	5	1	2	3	4	5	1	2	3	4	5
Adrenal glands – Subcapsular cell hyperplasia	1/4	3/4				3/4	1/4				2/3	1/3				2/4	1/4	1/4			3/4	1/4			
Urinary bladder – inflammatory cell foci											1/3					2/4	1/4								
Urinary bladder – urothelium vacuolation																1/4									
Testes – germ cell depletion	1/4					1/4															1/4				
Pancreas – inflammatory cell foci	1/4						1/4				1/3	2/3					2/4				2/4				
Pancreas – acinar cell degeneration											1/3														
Kidneys – hydronephrosis				1/4															2/4					3/4	
Kidneys – nephropathy											2/3					1/4	1/4				1/4				
Kidneys – inflammatory cell foci						1/4					1/3														
Kidneys – Cyst/s								1/4														1/4			
Lungs – B-adenoma	1/4																								
Skin – inflammatory cell foci											1/3										1/4				
Skin – scab formulation											1/3														
Liver – inflammatory cell foci																					2/4				
Ureter(s) - dilation																							1/4		
Epididymides – spermatic granuloma																							1/4		

Table S12. Microscopic observations during histopathological evaluation of organs/tissues of female mice sacrificed 43 weeks after the first injection. A 5-point grading scale was used: 1. Minimal change, 2. Mild change, 3. Moderate change, 4. Marked change, 5. Severe change.

Sex	**Females**																								
Group	Control group 1					Control group 2					Treatment group 1					Treatment group 2					Treatment group 3				
N	4					4					3					4					4				
Score	1	2	3	4	5	1	2	3	4	5	1	2	3	4	5	1	2	3	4	5	1	2	3	4	5
Adrenal glands – Subcapsular cell hyperplasia		4/4				1/4	2/4					2/3	1/3				3/4	1/4				4/4			
Urinary bladder – inflammatory cell foci											1/3					1/4									
Urinary bladder – urothelium vacuolation											1/3					1/4									
Urinary bladder – mineralization																						1/4			
Spleen – extramedullary haematopoiesis		1/4																							
Pancreas – inflammatory cell foci							1/4															1/4			
Kidneys – hydronephrosis																				1/4				1/4	1/4
Kidneys – nephropathy																			1/4						
Lungs – alveolar epithelialization																	1/4								
Lungs – B-adenoma																					1/4				
Liver – inflammatory cell foci						1/4										3/4									
Ureter(s) - dilation																							1/4		
Ovaries – atrophy														3/3					3/4					4/4	
Uterus – endometrial hyperplasia									1/4					3/3											

Table S13. Hematologic parameters in male mice. Values represent the mean ± SD. The reference values are based on the baseline (-1 wk) measurements. Asterisks show the significance level with *: $p \leq 0.05$.

$^{C1/C2/T1/T2/T3}$ Significant difference as compared with e.g. control group 1 (C1) at the same time point.

^ Outlier excluded from dataset (N = N-1)

Males	N	WBC ($10^3/mm^3$)	RBC ($10^6/mm^3$)	HGB (g/dl)	HCT (%)
Reference value	50	7.68 ± 1.85	9.21 ± 0.66	16.41 ± 0.84	44.49 ± 3.06
Control group 1: 5 wk	10	8.63 ± 1.77^{T3*}	8.92 ± 0.29^	16.52 ± 0.80	42.52 ± 1.44^
11 wk	7	8.38 ± 1.06^	9.40 ± 0.25	17.21 ± 0.41	44.11 ± 1.11
19 wk	7	11.17 ± 5.92	9.42 ± 0.30	16.80 ± 0.65	44.81 ± 1.69
30 wk	4	10.18 ± 3.47	9.21 ± 0.37	17.13 ± 0.85	44.30 ± 1.59
43 wk	4	5.98 ± 1.18	8.66 ± 0.19	15.63 ± 0.59	41.05 ± 0.74
Control group 2: 5 wk	10	7.67 ± 1.50	9.03 ± 0.65	16.45 ± 0.49	43.00 ± 3.33
11 wk	7	8.37 ± 2.67	9.13 ± 1.47	17.11 ± 0.85	44.96 ± 3.10
19 wk	7	9.53 ± 2.84	8.64 ± 0.42$^{T1*/T2*}$	15.59 ± 0.31^{T2*}	40.80 ± 1.97$^{T1*/T2*}$
30 wk	4	9.80 ± 2.25	9.35 ± 0.34	17.20 ± 0.43	44.90 ± 1.44
43 wk	4	6.70 ± 2.71	8.79 ± 0.34	15.38 ± 0.75	41.88 ± 1.83
Treatment group 1: 5 wk	10	8.08 ± 2.38	8.96 ± 0.30	16.78 ± 0.48^	42.96 ± 1.20
11 wk	7	8.53 ± 1.17	9.56 ± 0.79	17.49 ± 1.16	45.46 ± 3.66
19 wk	6	8.12 ± 1.98	9.58 ± 0.55^{C2*}	16.88 ± 0.38	45.67 ± 2.97^{C2*}
30 wk	3	9.77 ± 2.82	9.58 ± 0.27	16.93 ± 0.32	45.97 ± 2.38
43 wk	3	7.53 ± 0.83	8.90 ± 1.54	16.03 ± 2.27	42.70 ± 6.76
Treatment group 2: 5 wk	10	6.65 ± 2.80	8.75 ± 0.44	16.35 ± 0.65	42.99 ± 2.34
11 wk	7	9.61 ± 1.52	10.50 ± 1.43	18.46 ± 2.23	49.27 ± 7.70
19 wk	7	8.68 ± 0.34^	9.69 ± 0.91^{C2*}	17.44 ± 1.54^{C2*}	46.13 ± 4.88^{C2*}
30 wk	4	11.13 ± 3.50	9.14 ± 0.52	16.60 ± 0.10^	42.07 ± 0.45^
43 wk	4	7.30 ± 2.24	8.77 ± 0.41	15.63 ± 0.74	41.48 ± 2.44
Treatment group 3: 5 wk	10	5.48 ± 1.82^{C1*}	8.70 ± 0.35	16.02 ± 0.63	42.81 ± 2.14
11 wk	7	7.29 ± 1.54	9.26 ± 0.28	16.81 ± 0.43	43.09 ± 1.65
19 wk	7	8.40 ± 1.33	9.24 ± 0.34	16.63 ± 0.78	43.34 ± 2.21
30 wk	4	12.63 ± 3.38	9.31 ± 0.70	17.08 ± 0.94	44.23 ± 3.32
43 wk	4	7.68 ± 3.01	9.03 ± 0.35	16.10 ± 0.00^	42.80 ± 1.56

HCT: hematocrit; HGB: hemoglobin;; RBC: red blood cell count; WBC: white blood cell count.

Table S14. Hematologic parameters in male mice. Values represent the mean ± SD. The reference values are based on the baseline (-1 wk) measurements. Asterisks show the significance level with *: p ≤ 0.05.

$^{C1/C2/T1/T2/T3}$ Significant difference as compared with e.g. control group 1 (C1) at the same time point.

^ Outlier excluded from dataset (N = N-1)

Males	N	MCV (µm^3)	MCH (pg)	MCHC (g/dl)	PLT (10^3/mm^3)
Reference value	50	48.31 ± 0.68^	17.85 ± 0.55	36.93 ± 1.12	718.02 ± 175.49
Control group 1: 5 wk	10	47.70 ± 0.67^{T2*}	18.27 ± 0.47	38.29 ± 1.36	825.60 ± 121.58
11 wk	7	47.14 ± 0.69	18.33 ± 0.39	39.04 ± 0.82	744.71 ± 130.80
19 wk	7	47.43 ± 0.53	18.17 ± 0.35^	38.22 ± 0.81^	935.14 ± 164.96
30 wk	4	48.25 ± 0.50	18.58 ± 0.68	38.60 ± 1.29	1163.75 ± 168.54
43 wk	4	47.50 ± 0.58	18.17 ± 0.06^/$^{C2*/T3*}$	38.00 ± 0.85	1183.75 ± 141.96
Control group 2: 5 wk	10	47.50 ± 0.71$^{T2*/T3*}$	18.29 ± 1.12	38.44 ± 2.56	863.90 ± 120.87
11 wk	7	46.86 ± 0.90	18.20 ± 0.26^	38.60 ± 0.35^	738.14 ± 174.38
19 wk	7	47.43 ± 0.98	18.09 ± 0.79	38.11 ± 1.44	947.29 ± 198.53
30 wk	4	48.00 ± 0.82	18.43 ± 0.43	38.33 ± 1.04	1157.25 ± 52.10
43 wk	4	47.50 ± 0.58	17.48 ± 0.38$^{C1*/T1*}$	36.70 ± 0.89	1005.25 ± 118.46
Treatment group 1: 5 wk	10	48.10 ± 0.74	18.50 ± 0.58	38.54 ± 1.27	839.00 ± 102.63
11 wk	7	47.57 ± 0.98	18.31 ± 0.74	38.51 ± 1.74	719.00 ± 153.62
19 wk	6	47.20 ± 0.45^	17.67 ± 0.90	37.08 ± 2.28	929.50 ± 162.70
30 wk	3	47.67 ± 1.15	17.70 ± 0.70	37.00 ± 2.23	1110.33 ± 130.11
43 wk	3	48.00 ± 1.00	18.50 ± 0.00^/^{C2*}	37.70 ± 1.15	1133.67 ± 46.61
Treatment group 2: 5 wk	10	49.20 ± 1.23$^{C1*/C2*}$	18.68 ± 0.52	38.03 ± 1.64	870.44 ± 38.83^
11 wk	7	47.00 ± 1.00	17.63 ± 0.60	37.69 ± 1.71	693.57 ± 226.99
19 wk	7	47.57 ± 1.40	18.01 ± 0.46	38.47 ± 1.34^	873.17 ± 90.74^
30 wk	4	47.00 ± 0.00^	18.50 ± 0.57	39.13 ± 0.83	1086.25 ± 76.59
43 wk	4	47.50 ± 1.29	17.90 ± 0.00^	37.75 ± 1.19	1278.50 ± 89.12
Treatment group 3: 5 wk	10	48.67 ± 0.87^/^{C2*}	18.42 ± 0.83	37.48 ± 2.49	798.40 ± 121.37
11 wk	7	46.43 ± 0.79	18.14 ± 0.29	39.03 ± 0.67	830.00 ± 73.06^
19 wk	7	46.86 ± 1.21	17.99 ± 0.58	38.41 ± 1.24	917.86 ± 118.00
30 wk	4	48.00 ± 0.00^	18.38 ± 0.40	38.65 ± 0.85	955.50 ± 188.55
43 wk	4	48.00 ± 0.00^	17.60 ± 0.22$^{C1*/T1*}$	37.08 ± 0.53	1062.75 ± 90.16

MCH: mean cell hemoglobin; MCHC: mean cell hemoglobin concentration; MCV: mean cell volume; PLT: platelet count.

Table S15. Hematologic parameters in female mice. Values represent the mean ± SD. The reference values are based on the baseline (-1 wk) measurements. Asterisks show the significance level with *: p ≤ 0.05.
$^{C1/C2/T1/T2/T3}$ Significant difference as compared with e.g. control group 1 (C1) at the same time point.
^ Outlier excluded from dataset (N = N-1)

Females	N	WBC ($10^3/mm^3$)	RBC ($10^6/mm^3$)	HGB (g/dl)	HCT (%)
Reference value	50	7.63 ± 2.25	8.53 ± 0.57^	15.75 ± 0.75	41.69 ± 2.64
Control group 1: 5 wk	10	7.15 ± 0.94^{T3*}	8.36 ± 0.57^	15.76 ± 0.96	40.88 ± 2.65^
11 wk	7	7.71 ± 2.53	9.19 ± 0.38	17.00 ± 0.19^	43.61 ± 1.51
19 wk	7	7.63 ± 0.51^	9.07 ± 0.50	17.00 ± 0.81	42.86 ± 2.27
30 wk	4	9.03 ± 1.48	9.34 ± 0.19	17.00 ± 0.00$^{\wedge/T3}$	44.90 ± 1.64
43 wk	4	8.30 ± 3.68	8.94 ± 0.47	16.00 ± 0.50	42.65 ± 1.82
Control group 2: 5 wk	10	7.27 ± 1.51^{T3*}	8.42 ± 0.39	15.61 ± 0.74^	40.85 ± 3.14
11 wk	7	8.51 ± 2.34	9.01 ± 0.34	16.27 ± 0.49	42.01 ± 1.60
19 wk	7	6.74 ± 3.19	8.57 ± 0.55	15.97 ± 1.00	40.57 ± 2.81
30 wk	4	10.38 ± 2.86	9.30 ± 0.44	16.53 ± 0.51	44.18 ± 2.83
43 wk	4	7.40 ± 0.72^	8.92 ± 0.63	16.38 ± 1.01	42.08 ± 3.01
Treatment group 1: 5 wk	10	6.20 ± 2.24	8.51 ± 0.45	16.19 ± 0.58	41.51 ± 2.18
11 wk	7	7.60 ± 3.77	8.59 ± 0.31^	15.88 ± 0.43^	40.26 ± 1.41
19 wk	7	6.80 ± 1.64	9.37 ± 1.10	16.90 ± 1.83	43.89 ± 5.14
30 wk	4	9.70 ± 2.96	8.71 ± 0.27	16.30 ± 0.52	41.33 ± 0.79
43 wk	3	7.20 ± 3.97	8.91 ± 0.89	16.50 ± 0.66	43.27 ± 3.17
Treatment group 2: 5 wk	10	5.89 ± 2.56	8.70 ± 0.63	16.27 ± 0.78	41.91 ± 3.22
11 wk	7	7.97 ± 2.09	9.05 ± 0.72	16.59 ± 0.85	41.64 ± 3.22
19 wk	7	6.74 ± 1.77	8.61 ± 0.98	15.59 ± 1.56	39.66 ± 4.17
30 wk	4	8.98 ± 1.67	9.07 ± 0.27	16.65 ± 0.31	43.50 ± 1.68
43 wk	4	9.10 ± 3.17	8.96 ± 0.18	15.53 ± 0.85	41.90 ± 1.79
Treatment group 3: 5 wk	10	4.36 ± 1.67$^{C1*/C2*}$	8.75 ± 0.47	16.04 ± 0.44	42.16 ± 2.40
11 wk	7	7.57 ± 1.58	9.04 ± 0.32^	16.56 ± 0.81	43.04 ± 2.97
19 wk	7	7.69 ± 2.31	9.62 ± 1.71	16.44 ± 1.77	44.93 ± 7.28
30 wk	4	7.93 ± 2.49	9.22 ± 0.66	15.83 ± 0.06$^{\wedge/C1*}$	41.13 ± 0.23^
43 wk	4	8.90 ± 3.34	9.72 ± 0.26	15.30 ± 0.29	44.95 ± 1.05

HCT: hematocrit; HGB: hemoglobin;; RBC: red blood cell count; WBC: white blood cell count.

Table S16. Hematologic parameters in female mice. Values represent the mean ± SD. The reference values are based on the baseline (-1 wk) measurements. Asterisks show the significance level with *: $p \leq 0.05$.
$^{C1/C2/T1/T2/T3}$ Significant difference as compared with e.g. control group 1 (C1) at the same time point.
^ Outlier excluded from dataset (N = N-1)

Females	N	MCV (μm^3)	MCH (pg)	MCHC (g/dl)	PLT ($10^3/mm^3$)
Reference value	50	48.78 ± 0.65	18.46 ± 0.63	37.77 ± 1.09	665.68 ± 177.78
Control group 1: 5 wk	10	49.10 ± 0.99$^{C2*/T3*}$	18.80 ± 0.44	38.80 ± 0.77^	828.80 ± 209.20
11 wk	7	47.43 ± 0.79^{T2*}	18.34 ± 0.36	38.60 ± 0.55	872.71 ± 104.41
19 wk	7	47.29 ± 0.76	18.76 ± 0.41^{T3*}	39.71 ± 0.84^{T3*}	910.14 ± 153.55
30 wk	4	48.00 ± 0.82	17.98 ± 0.68	37.50 ± 2.07	966.25 ± 201.80
43 wk	4	48.00 ± 0.82	17.95 ± 1.12	37.55 ± 1.74^{T3*}	1041.25 ± 87.21
Control group 2: 5 wk	10	47.50 ± 0.71^{C1*}	18.55 ± 0.34	39.02 ± 0.47	949.60 ± 59.20^{T2*}
11 wk	7	46.57 ± 0.53	18.07 ± 0.29	38.77 ± 0.49	839.00 ± 244.75
19 wk	7	47.14 ± 0.69	18.67 ± 0.50^{T3*}	39.43 ± 1.37	910.29 ± 143.31
30 wk	4	47.75 ± 0.96	17.80 ± 0.91	37.55 ± 2.64	982.75 ± 135.76
43 wk	4	47.00 ± 0.00^	18.35 ± 0.19^{T3*}	38.88 ± 0.59^{T3*}	854.75 ± 343.71
Treatment group 1: 5 wk	10	48.33 ± 0.71^	19.04 ± 0.44^{T3*}	39.05 ± 1.35	774.80 ± 109.24
11 wk	7	46.71 ± 0.95	18.50 ± 0.71	39.76 ± 1.01	722.14 ± 162.36
19 wk	7	46.86 ± 0.69	18.37 ± 0.28^	38.43 ± 1.45	792.14 ± 187.73
30 wk	4	47.00 ± 0.00^	18.75 ± 0.99	39.45 ± 1.74	1011.67 ± 28.22^
43 wk	3	49.00 ± 0.00^	18.60 ± 1.08^{T3*}	38.30 ± 1.30^{T3*}	675.33 ± 129.51
Treatment group 2: 5 wk	10	48.10 ± 0.74	18.74 ± 0.80	38.91 ± 1.70	698.60 ± 200.39^{C2*}
11 wk	7	45.86 ± 0.69^{C1*}	18.37 ± 0.74	39.93 ± 1.51	798.71 ± 116.74
19 wk	7	46.00 ± 1.15	18.16 ± 0.81	39.31 ± 1.26	806.14 ± 175.91
30 wk	4	48.00 ± 0.00^	17.93 ± 0.06^	37.27 ± 0.23^	1028.00 ± 41.41
43 wk	4	46.75 ± 2.06	17.33 ± 1.17	37.05 ± 1.17^{T3*}	952.00 ± 199.80
Treatment group 3: 5 wk	10	48.00 ± 0.82^{C1*}	18.25 ± 0.66^{T1*}	38.14 ± 1.64	764.67 ± 72.21^
11 wk	7	46.43 ± 0.98	17.90 ± 0.63	38.56 ± 1.23	731.29 ± 144.20
19 wk	7	46.86 ± 1.77	17.29 ± 1.56$^{C1*/C2*}$	36.94 ± 2.97^{C1*}	742.14 ± 181.84
30 wk	4	46.50 ± 1.29	17.75 ± 0.42	38.50 ± 0.10^	996.50 ± 96.63
43 wk	4	46.25 ± 0.96	15.90 ± 0.00$^{\wedge/C2*/T1*}$	34.08 ± 0.28$^{C1*/C2*/T1*/T2*}$	763.00 ± 260.79

MCH: mean cell hemoglobin; MCHC: mean cell hemoglobin concentration; MCV: mean cell volume; PLT: platelet count.

Table S17. Clinical chemistry parameters in male mice. Values represent the mean ± SD. The reference values are based on the baseline (-1 wk) measurements. Asterisks show the significance level with *: $p \leq 0.05$.

C1/C2/T1/T2/T3 Significant difference as compared with e.g. control group 1 (C1) at the same time point.

^ Outlier excluded from dataset (N = N-1)

		Electrolytes			*Ions*	*Small molecules (nutrition)*	*Small molecules (waste products)*	
**Males**	**N**	Sodium (mmol/L)	Potassium (mmol/L)	Chloride (mmol/L)	Calcium (mmol/L)	Glucose (mmol/L)	Creatinine (µmol/L)	Urea (mmol/L)
Reference value	50	166.92 ± 3.52	8.59 ± 0.89	111.34 ± 4.66	2.75 ± 0.10	8.27 ± 1.33	45.30 ± 11.08	6.95 ± 0.85
Control group 1: 5 wk	10	149.30 ± 4.22	6.38 ± 0.84	98.90 ± 6.17	2.36 ± 0.06	5.74 ± 1.24	32.00 ± 12.90	7.05 ± 1.00
11 wk	7	158.57 ± 2.07	7.24 ± 0.32	100.29 ± 1.60	2.37 ± 0.04	5.91 ± 0.64	51.00 ± 19.67	6.39 ± 0.73
19 wk	4	142.50 ± 2.89	6.63 ± 0.48	100.00 ± 4.08	1.99 ± 0.09	5.50 ± 0.58	ND	5.75 ± 0.65
30 wk	4	168.75 ± 4.79	7.00 ± 0.41	111.25 ± 4.79	2.31 ± 0.07	6.50 ± 0.41	21.25 ± 11.81	6.38 ± 0.85
43 wk	4	150.00 ± 0.00	5.50 ± 0.41	100.00 ± 0.00	1.98 ± 0.14	6.38 ± 0.85	ND	8.50 ± 2.12
Control group 2: 5 wk	10	149.10 ± 3.70	6.71 ± 1.10	99.90 ± 7.28	2.38 ± 0.10	6.69 ± 1.54	32.90 ± 16.20	7.17 ± 0.64^
11 wk	7	158.14 ± 1.46	7.33 ± 0.29	100.71 ± 2.93	2.36 ± 0.02^	7.41 ± 2.00	50.14 ± 21.47	5.61 ± 0.86 T2*
19 wk	4	140.00 ± 0.00 T2*	6.63 ± 0.48	100.00 ± 0.00^	1.96 ± 0.09	5.50 ± 0.00^	ND	5.75 ± 0.65
30 wk	4	165.00 ± 0.00	7.00 ± 0.41	110.00 ± 0.00	2.38 ± 0.12	7.63 ± 0.48	36.25 ± 16.52	6.75 ± 1.55
43 wk	4	151.25 ± 6.29	5.38 ± 0.63	100.00 ± 0.00^	1.98 ± 0.03	6.50 ± 1.47	ND	8.00 ± 2.12
Treatment group 1: 5 wk	10	148.22 ± 3.56^	6.70 ± 1.10	100.00 ± 6.83	2.33 ± 0.05	5.77 ± 1.70	32.80 ± 14.64	7.44 ± 0.85
11 wk	7	158.50 ± 2.95^	7.50 ± 0.55	101.00 ± 1.55^	2.35 ± 0.11	6.56 ± 0.84	70.00 ± 5.25^	6.60 ± 0.60
19 wk	3	145.00 ± 0.00^	6.50 ± 0.00^	100.00 ± 0.00	2.05 ± 0.00^	5.17 ± 0.76	ND	7.00 ± 0.00^
30 wk	3	170.00 ± 0.00	7.00 ± 0.00^	110.00 ± 0.00^	2.37 ± 0.08	6.83 ± 1.04	35.00 ± 13.23	7.50 ± 0.00
43 wk	3	150.00 ± 0.00^	6.00 ± 0.00^	100.00 ± 0.00^	2.02 ± 0.10	6.17 ± 1.89	ND	7.50 ± 0.00^
Treatment group 2: 5 wk	10	150.11 ± 2.32^	6.26 ± 0.79	100.30 ± 5.87	2.38 ± 0.10	5.13 ± 1.11	35.70 ± 11.01	7.46 ± 1.14
11 wk	7	160.71 ± 2.36	7.25 ± 0.23^	103.71 ± 2.36	2.37 ± 0.03^	5.31 ± 1.34	ND	7.24 ± 1.41 C2*
19 wk	4	147.50 ± 2.89 C2*	6.50 ± 0.00^	105.00 ± 0.00^	2.06 ± 0.09	5.75 ± 0.65	ND	7.13 ± 0.85
30 wk	4	165.00 ± 0.00^	7.25 ± 0.29	110.00 ± 0.00^	2.25 ± 0.00^	6.63 ± 2.10	42.50 ± 25.98	8.00 ± 1.35
43 wk	4	150.00 ± 4.08	6.75 ± 2.06	100.00 ± 0.00^	1.98 ± 0.13	5.25 ± 0.87	ND	7.33 ± 1.26^
Treatment group 3: 5 wk	10	150.20 ± 4.44	6.27 ± 0.75	99.80 ± 8.98	2.36 ± 0.07	5.60 ± 2.45	31.50 ± 16.08	7.59 ± 1.51
11 wk	7	159.00 ± 2.45	7.24 ± 0.50	102.00 ± 2.45	2.42 ± 0.08	6.09 ± 1.80	ND	6.77 ± 0.71
19 wk	4	143.75 ± 4.79	6.25 ± 0.29	100.00 ± 0.00^	2.05 ± 0.14	4.75 ± 0.87	ND	7.25 ± 0.87
30 wk	4	166.25 ± 6.29	7.00 ± 0.00^	110.00 ± 4.08	2.30 ± 0.11	6.88 ± 1.89	30.00 ± 15.81	6.50 ± 0.00^
43 wk	4	150.00 ± 4.08	5.13 ± 0.63	100.00 ± 0.00^	2.00 ± 0.00	6.13 ± 1.65	ND	6.75 ± 1.04

ND: Not determined. (Value was below detection limit.)

Table S18. Clinical chemistry parameters in male mice. Values represent the mean ± SD. The reference values are based on the baseline (-1 wk) measurements. Asterisks show the significance level with *: $p \leq 0.05$.

$^{C1/C2/T1/T2/T3}$ Significant difference as compared with e.g. control group 1 (C1) at the same time point.

^ Outlier excluded from dataset (N = N-1)

Males	N	*Proteins (general)* Total Protein (g/L)	*Proteins (enzymes)* ALT (U/L)	AST (U/L)	Alk. Phosphatase (U/L)	Amylase (U/L)	Lipase (U/L)	*Lipids* Cholesterol (mmol/L)
Reference value	50	62.70 ± 3.11	37.84 ± 10.74^	221.33 ± 152.18^	202.78 ± 21.39^	3821.14 ± 922.61^	34.80 ± 4.16	3.60 ± 0.32
Control group 1: 5 wk	10	48.50 ± 3.21	38.22 ± 6.14^	86.90 ± 21.42^{T1*}	120.44 ± 8.59^$^{/T3*}$	2537.80 ± 263.31	27.20 ± 2.82	3.00 ± 0.22
11 wk	7	54.00 ± 0.00^	42.00 ± 6.00	136.29 ± 55.96	98.14 ± 8.25	2586.00 ± 69.30^	29.57 ± 2.70	3.00 ± 0.00^
19 wk	4	42.50 ± 6.45	ND	88.75 ± 11.09	70.00 ± 0.00^$^{/T2*/T3*}$	2075.00 ± 37.75^	12.50 ± 6.45	2.50 ± 0.00^
30 wk	4	50.00 ± 4.08^{C2*}	23.83 ± 0.29^	110.00 ± 25.50	72.50 ± 6.45	2667.50 ± 251.35	33.75 ± 6.29	2.50 ± 0.00^
43 wk	4	45.00 ± 4.08	58.75 ± 17.97	81.25 ± 30.92	72.50 ± 6.45	2458.75 ± 46.08 ^{T1*}	25.00 ± 0.00^	2.50 ± 0.41
Control group 2: 5 wk	10	48.60 ± 1.78	50.20 ± 16.17	119.56 ± 53.82^	117.70 ± 8.30	2488.67 ± 189.94^	29.20 ± 3.68^{T3*}	2.84 ± 0.15^
11 wk	7	54.86 ± 1.46	40.71 ± 7.32	111.50 ± 30.02^	100.29 ± 7.72	2499.00 ± 138.83^	25.29 ± 2.93	2.87 ± 0.24
19 wk	4	46.25 ± 4.79	ND	158.75 ± 58.65	62.50 ± 6.45$^{T2*/T3**}$	2016.25 ± 219.79	20.00 ± 7.07	2.38 ± 0.48
30 wk	4	55.00 ± 0.00^{C1*}	31.25 ± 6.29	136.25 ± 53.44	80.00 ± 7.07	2700.00 ± 135.83	32.50 ± 6.45	2.75 ± 0.29
43 wk	4	45.00 ± 0.00^	50.00 ± 16.83	65.00 ± 19.58	67.50 ± 2.89	2427.50 ± 167.21^{T1*}	25.00 ± 0.00^	2.50 ± 0.00^
Treatment group 1: 5 wk	10	49.40 ± 2.41	48.44 ± 11.20^	158.70 ± 85.00 ^{C1*}	114.00 ± 8.28^	2539.56 ± 142.08^	27.07 ± 4.24^	3.02 ± 0.23
11 wk	7	53.57 ± 3.64^{T3*}	42.00 ± 4.58	149.57 ± 51.97	98.57 ± 4.04	2644.00 ± 166.86^$^{/T2*}$	30.50 ± 2.26^	2.87 ± 0.24
19 wk	3	40.00 ± 0.00^	ND	93.33 ± 23.63	70.00 ± 0.00^	2098.33 ± 102.63	25.00 ± 13.23	2.50 ± 0.00^
30 wk	3	55.00 ± 0.00^	25.00 ± 0.00^	80.00 ± 22.91	85.00 ± 10.00	3003.33 ± 122.51	35.00 ± 5.00	2.50 ± 0.00^
43 wk	3	45.00 ± 0.00^	40.00 ± 0.00^	113.33 ± 27.57	68.33 ± 20.82	2970.00 ± 0.00^$^{/C1*/C2*/T3*}$	36.67 ± 12.58	2.50 ± 0.00
Treatment group 2: 5 wk	10	51.20 ± 3.26	43.67 ± 9.67^	146.40 ± 38.81	112.40 ± 16.22	2535.80 ± 223.82	24.98 ± 4.66	3.13 ± 0.41
11 wk	7	56.14 ± 2.27	36.86 ± 2.85	117.00 ± 41.82	96.86 ± 9.44	2424.43 ± 133.11^{T1*}	25.71 ± 2.93	3.04 ± 0.21
19 wk	4	45.00 ± 0.00^	ND	106.25 ± 56.77	85.00 ± 0.00^$^{/C1*/C2*}$	2172.50 ± 97.77	18.75 ± 8.54	2.50 ± 0.00
30 wk	4	50.00 ± 0.00^	32.63 ± 8.42	152.50 ± 72.63	80.00 ± 0.00	2852.50 ± 169.53	35.00 ± 13.54	2.50 ± 0.00
43 wk	4	47.50 ± 2.89	53.75 ± 20.56	93.75 ± 8.54	75.00 ± 15.81	2566.25 ± 206.74	33.75 ± 11.81	2.50 ± 0.00^
Treatment group 3: 5 wk	10	49.50 ± 2.42	46.70 ± 13.01	116.80 ± 47.99	103.20 ± 13.73^{C1*}	2547.22 ± 278.84^	22.50 ± 3.27^{C2*}	3.15 ± 0.34
11 wk	7	57.43 ± 2.07^{T1*}	44.14 ± 4.14	102.86 ± 34.63	99.43 ± 5.59	2533.50 ± 45.96^	27.00 ± 4.90	3.04 ± 0.27
19 wk	4	48.75 ± 6.29	ND	88.75 ± 40.29	83.75 ± 7.50$^{C1*/C2**}$	2346.67 ± 32.53^	23.75 ± 7.50	2.50 ± 0.41
30 wk	4	50.00 ± 0.00^	30.00 ± 0.00^	117.50 ± 32.02	87.50 ± 8.66	2842.50 ± 114.20	30.00 ± 7.07	2.75 ± 0.29
43 wk	4	45.00 ± 0.00^	63.75 ± 24.96	97.50 ± 49.41	80.00 ± 17.80	2453.75 ± 188.74^{T1*}	30.00 ± 7.07	2.50 ± 0.00^

ND: Not determined. (Value was below detection limit.)

Table S19. Clinical chemistry parameters in female mice. Values represent the mean ± SD. The reference values are based on the baseline (-1 wk) measurements. Asterisks show the significance level with *: $p \leq 0.05$.
$^{C1/C2/T1/T2/T3}$ Significant difference as compared with e.g. control group 1 (C1) at the same time point.
^ Outlier excluded from dataset (N = N-1)

		Electrolytes			*Ions*	*Small molecules (nutrition)*	*Small molecules (waste products)*	
**Females**	**N**	Sodium (mmol/L)	Potassium (mmol/L)	Chloride (mmol/L)	Calcium (mmol/L)	Glucose (mmol/L)	Creatinine (µmol/L)	Urea (mmol/L)
Reference value	25	172.32 ± 4.25	8.18 ± 0.80	106.68 ± 2.75	2.61 ± 0.12	7.24 ± 0.76	51.64 ± 10.42	7.41 ± 1.11
Control group 1: 5 wk	10	142.20 ± 3.22$^{T2**/T3**}$	9.75 ± 1.76	102.30 ± 2.63$^{T2*/T3*}$	2.24 ± 0.07	5.10 ± 1.16	39.30 ± 3.59	7.71 ± 1.32^{T2*}
11 wk	7	150.00 ± 0.00	7.11 ± 0.79	99.00 ± 0.00	2.29 ± 0.07	4.76 ± 0.59	69.86 ± 13.93	6.20 ± 0.73^
19 wk	7	142.86 ± 4.88	5.71 ± 0.76	100.71 ± 3.45	2.13 ± 0.11	5.50 ± 1.12	ND	6.29 ± 0.95^{T3**}
30 wk	4	167.50 ± 2.89	6.75 ± 0.29	110.00 ± 0.00	2.34 ± 0.06	6.38 ± 0.48	40.00 ± 14.72	6.38 ± 0.85
43 wk	4	150.00 ± 0.00	5.00 ± 0.41	105.00 ± 0.00^$^{/T1*/T2*}$	2.01 ± 0.07	5.88 ± 1.31	37.00 ± 1.73^	6.50 ± 0.00^
Control group 2: 5 wk	10	144.60 ± 3.10	8.52 ± 1.83	104.10 ± 2.47^{T2*}	2.22 ± 0.06	4.95 ± 1.06	38.70 ± 4.79	7.83 ± 0.88^{T2*}
11 wk	7	145.29 ± 7.52$^{T2*/T3*}$	7.07 ± 0.62	97.29 ± 6.21	2.24 ± 0.13^{T2*}	5.40 ± 1.04	69.86 ± 25.96	7.07 ± 1.19
19 wk	7	147.14 ± 2.67	5.71 ± 1.15	103.57 ± 2.44	2.11 ± 0.13	5.79 ± 0.39	ND	6.14 ± 0.38^{T3**}
30 wk	4	165.00 ± 4.08	6.75 ± 0.29	112.50 ± 2.89	2.35 ± 0.06	6.50 ± 1.08	23.75 ± 7.50	7.00 ± 0.00^
43 wk	4	145.00 ± 5.00^	4.38 ± 1.03	101.67 ± 2.89^	2.02 ± 0.06^	5.00 ± 2.08	28.00 ± 4.24	6.17 ± 0.76^
Treatment group 1: 5 wk	10	142.50 ± 2.92$^{T2**/T3*}$	9.09 ± 1.84	102.60 ± 3.10$^{T2*/T3*}$	2.22 ± 0.07	4.50 ± 1.49	37.20 ± 9.61	6.84 ± 1.27$^{T2***/T3**}$
11 wk	7	150.00 ± 0.00	7.37 ± 0.45	100.71 ± 1.60	2.27 ± 0.08	5.96 ± 0.74^{T2*}	56.14 ± 22.30	7.24 ± 1.10
19 wk	7	142.86 ± 2.67	5.64 ± 0.94	102.86 ± 2.67	2.14 ± 0.12	5.93 ± 1.06	ND	7.29 ± 1.04
30 wk	4	165.00 ± 0.00^	7.50 ± 0.00^	111.25 ± 4.79	2.33 ± 0.03	6.50 ± 1.22	37.50 ± 24.66	7.00 ± 1.78
43 wk	3	145.00 ± 0.00^	5.00 ± 0.00^	100.00 ± 0.00^$^{/C1*/T3*}$	2.05 ± 0.00^	6.83 ± 1.04	33.00 ± 0.00^	6.00 ± 0.00^
Treatment group 2: 5 wk	10	147.90 ± 2.47$^{C1**/T1**}$	8.04 ± 1.38	108.30 ± 2.63$^{C1*/C2*/T1*}$	2.28 ± 0.07	5.91 ± 1.17	36.30 ± 3.59	9.48 ± 0.83$^{C1*/C2*/T1***}$
11 wk	7	152.57 ± 4.72^{C2*}	7.33 ± 0.62	100.71 ± 3.82	2.40 ± 0.10^{C2*}	4.60 ± 0.41^$^{/T1*}$	73.29 ± 19.12	7.41 ± 0.62
19 wk	7	143.57 ± 3.78	5.71 ± 1.04	102.14 ± 2.67	2.10 ± 0.16	6.00 ± 0.82	ND	6.64 ± 1.07^{T3*}
30 wk	4	165.00 ± 0.00^	7.38 ± 0.48	110.00 ± 0.00^	2.41 ± 0.05	7.13 ± 0.48	61.25 ± 18.87	7.50 ± 0.71
43 wk	4	150.00 ± 7.07	5.25 ± 0.65	100.00 ± 0.00^$^{/C1*/T3*}$	2.15 ± 0.20	5.88 ± 0.95	34.00 ± 9.59	8.13 ± 2.95
Treatment group 3: 5 wk	10	147.30 ± 4.11$^{C1**/T1*}$	8.19 ± 1.94	107.10 ± 3.75$^{C1*/T1*}$	2.25 ± 0.08	5.28 ± 1.42	35.40 ± 4.86	8.94 ± 1.32^{T1**}
11 wk	7	153.00 ± 3.46^{C2*}	7.67 ± 0.52	101.14 ± 2.27	2.37 ± 0.08	5.57 ± 1.02	72.00 ± 25.40	7.97 ± 1.26
19 wk	7	143.57 ± 2.44	5.86 ± 0.90	102.86 ± 2.67	2.08 ± 0.08	6.43 ± 1.17	ND	8.50 ± 1.80$^{C1**/C2**/T2*}$
30 wk	4	167.50 ± 2.89	7.13 ± 0.48	112.50 ± 2.89	2.39 ± 0.05	7.00 ± 0.41	37.50 ± 22.17	9.25 ± 2.40
43 wk	4	150.00 ± 0.00^	5.75 ± 1.19	105.00 ± 0.00^$^{/T1*/T2*}$	2.10 ± 0.05^	9.50 ± 4.38	36.75 ± 7.09	8.63 ± 2.56

ND: Not determined. (Value was below detection limit.)

Table S20. Clinical chemistry parameters in female mice. Values represent the mean ± SD. The reference values are based on the baseline (-1 wk) measurements. Asterisks show the significance level with *: $p \leq 0.05$.
$^{C1/C2/T1/T2/T3}$ Significant difference as compared with e.g. control group 1 (C1) at the same time point.
^ Outlier excluded from dataset (N = N-1)

		Proteins (general)	*Proteins (enzymes)*					*Lipids*
**Females**	**N**	Total Protein (g/L)	ALT (U/L)	AST (U/L)	Alk. Phosphatase (U/L)	Amylase (U/L)	Lipase (U/L)	Cholesterol (mmol/L)
Reference value	25	56.72 ± 3.12	36.36 ± 10.22	236.08 ± 104.44^	187.17 ± 21.50^	3043.58 ± 362.10^	33.32 ± 3.29	2.94 ± 0.28
Control group 1: 5 wk	10	48.30 ± 3.30	34.00 ± 15.66^	137.40 ± 57.25	103.67 ± 4.27^	2264.10 ± 320.44	25.50 ± 3.54$^{T2*/T3*}$	2.43 ± 0.22
11 wk	7	51.86 ± 1.46	33.00 ± 9.64	142.50 ± 56.63^	94.71 ± 9.93	2343.50 ± 56.65^	24.00 ± 2.45	2.19 ± 0.23
19 wk	7	47.14 ± 5.67	ND	100.00 ± 39.48	73.57 ± 5.56	2177.14 ± 88.59	22.86 ± 15.24	1.79 ± 0.27
30 wk	4	55.00 ± 0.00^	25.38 ± 3.50	115.00 ± 12.91	80.00 ± 12.25	2872.50 ± 241.95	35.00 ± 4.08	2.00 ± 0.00^
43 wk	4	45.00 ± 0.00^	52.50 ± 16.58	105.00 ± 30.28	77.50 ± 9.57	2368.75 ± 192.67	35.00 ± 5.77	1.75 ± 0.29
Control group 2: 5 wk	10	48.00 ± 2.00	35.33 ± 15.06^	159.90 ± 75.14	100.20 ± 6.96	2394.00 ± 456.64	23.70 ± 1.70	2.46 ± 0.13
11 wk	7	49.29 ± 2.93^{T2*}	33.00 ± 4.58	156.43 ± 55.17	84.43 ± 7.63	2479.00 ± 508.35^	22.71 ± 2.36	2.23 ± 0.24
19 wk	7	45.71 ± 6.07	ND	113.57 ± 40.28	73.57 ± 9.88	2310.00 ± 387.62	17.14 ± 8.09	1.86 ± 0.38
30 wk	4	52.50 ± 2.89	23.00 ± 0.87^	97.50 ± 17.56	86.25 ± 8.54	2881.25 ± 244.76	30.00 ± 0.00^	2.00 ± 0.00
43 wk	4	41.25 ± 11.09	38.75 ± 8.54	87.50 ± 22.55	71.25 ± 14.93^{T3*}	2196.25 ± 660.32	32.50 ± 8.66	2.00 ± 0.00^
Treatment group 1: 5 wk	10	47.70 ± 3.86	30.67 ± 7.76^	146.33 ± 50.91^	104.70 ± 11.61	2207.00 ± 90.54^	24.60 ± 2.76^{T2*}	2.52 ± 0.15
11 wk	7	50.14 ± 1.46^{T2*}	31.71 ± 8.64	168.43 ± 97.85	85.71 ± 8.98	2291.50 ± 234.84^	22.71 ± 5.44	2.23 ± 0.24
19 wk	7	45.00 ± 5.00	ND	93.57 ± 26.41	68.75 ± 4.76	2194.29 ± 115.59	18.57 ± 10.69	1.79 ± 0.27
30 wk	4	50.00 ± 0.00^	26.75 ± 10.37	177.50 ± 166.76	75.00 ± 0.00^	2801.25 ± 269.86	32.50 ± 2.89	2.00 ± 0.00^
43 wk	3	45.00 ± 5.00	80.00 ± 27.84	98.33 ± 10.41	86.67 ± 16.07	2558.33 ± 396.78	30.00 ± 0.00^	1.50 ± 0.00^
Treatment group 2: 5 wk	10	48.60 ± 3.95	39.67 ± 16.14^	166.00 ± 42.43^	96.60 ± 11.73	2298.30 ± 208.47	20.10 ± 4.01$^{C1*/T1*}$	2.67 ± 0.26
11 wk	7	54.00 ± 3.00$^{C2*/T1*}$	32.57 ± 8.90	168.00 ± 92.01	92.57 ± 8.90	2444.50 ± 166.64^	22.71 ± 2.93	2.49 ± 0.29
19 wk	7	43.57 ± 6.27	ND	121.43 ± 62.70	75.00 ± 8.16	2123.57 ± 314.48	16.43 ± 9.88	1.86 ± 0.24
30 wk	4	52.50 ± 2.89	31.88 ± 9.44	203.75 ± 113.31	87.50 ± 8.66	2660.00 ± 124.30	43.75 ± 4.79	2.50 ± 0.00^
43 wk	4	46.25 ± 6.29	65.00 ± 30.00	96.25 ± 34.73	88.75 ± 7.50	2225.00 ± 21.79^	33.75 ± 4.79	2.00 ± 0.00^
Treatment group 3: 5 wk	10	48.30 ± 4.11	38.33 ± 12.53	192.00 ± 67.84	96.00 ± 15.81	2403.60 ± 343.28	21.00 ± 4.69^{C1*}	2.47 ± 0.13^
11 wk	7	51.00 ± 3.00	38.57 ± 10.88	179.14 ± 76.54	93.00 ± 4.65^	2658.00 ± 343.28	25.29 ± 5.15	2.49 ± 0.33
19 wk	7	41.43 ± 2.44	ND	124.29 ± 33.59	81.43 ± 10.69	2228.57 ± 261.88	16.43 ± 10.69	2.00 ± 0.29
30 wk	4	52.50 ± 2.89	28.75 ± 8.33	156.25 ± 76.63	90.00 ± 9.13	2912.50 ± 427.40	36.25 ± 10.31	2.38 ± 0.48
43 wk	4	45.00 ± 0.00^	80.00 ± 27.99	135.00 ± 64.16	110.00 ± 26.46^{C2*}	2690.00 ± 667.66	45.00 ± 0.00^	2.00 ± 0.71

ND: Not determined. (Value was below detection limit.)
